# A score to predict and stratify risk of tuberculosis in adult contacts of tuberculosis index cases: a prospective derivation and external validation cohort study

**DOI:** 10.1016/S1473-3099(17)30447-4

**Published:** 2017-08-18

**Authors:** Matthew J Saunders, Tom Wingfield, Marco A Tovar, Matthew R Baldwin, Sumona Datta, Karine Zevallos, Rosario Montoya, Teresa R Valencia, Jon S Friedland, Larry H Moulton, Robert H Gilman, Carlton A Evans

**Affiliations:** Section of Infectious Diseases and Immunity, Imperial College London, and Wellcome Trust Imperial College Centre for Global Health Research, London, UK; Innovation for Health and Development (IFHAD), Laboratory of Research and Development, Universidad Peruana Cayetano Heredia, Lima, Peru; Innovación Por la Salud Y Desarrollo (IPSYD), Asociación Benéfica PRISMA, Lima, Peru; Section of Infectious Diseases and Immunity, Imperial College London, and Wellcome Trust Imperial College Centre for Global Health Research, London, UK; Innovation for Health and Development (IFHAD), Laboratory of Research and Development, Universidad Peruana Cayetano Heredia, Lima, Peru; Innovación Por la Salud Y Desarrollo (IPSYD), Asociación Benéfica PRISMA, Lima, Peru; Institute for Infection and Global Health, University of Liverpool, Liverpool, UK; Tropical and Infectious Diseases Unit, Royal Liverpool and Broadgreen University Hospitals Trust, Liverpool, UK; Innovation for Health and Development (IFHAD), Laboratory of Research and Development, Universidad Peruana Cayetano Heredia, Lima, Peru; Innovación Por la Salud Y Desarrollo (IPSYD), Asociación Benéfica PRISMA, Lima, Peru; Innovation for Health and Development (IFHAD), Laboratory of Research and Development, Universidad Peruana Cayetano Heredia, Lima, Peru; Innovación Por la Salud Y Desarrollo (IPSYD), Asociación Benéfica PRISMA, Lima, Peru; Columbia University, College of Physicians and Surgeons, New York, NY, USA; Section of Infectious Diseases and Immunity, Imperial College London, and Wellcome Trust Imperial College Centre for Global Health Research, London, UK; Innovation for Health and Development (IFHAD), Laboratory of Research and Development, Universidad Peruana Cayetano Heredia, Lima, Peru; Innovación Por la Salud Y Desarrollo (IPSYD), Asociación Benéfica PRISMA, Lima, Peru; Innovation for Health and Development (IFHAD), Laboratory of Research and Development, Universidad Peruana Cayetano Heredia, Lima, Peru; Innovación Por la Salud Y Desarrollo (IPSYD), Asociación Benéfica PRISMA, Lima, Peru; Innovación Por la Salud Y Desarrollo (IPSYD), Asociación Benéfica PRISMA, Lima, Peru; Innovation for Health and Development (IFHAD), Laboratory of Research and Development, Universidad Peruana Cayetano Heredia, Lima, Peru; Section of Infectious Diseases and Immunity, Imperial College London, and Wellcome Trust Imperial College Centre for Global Health Research, London, UK; Johns Hopkins Bloomberg School of Public Health, Baltimore, MD, USA; Section of Infectious Diseases and Immunity, Imperial College London, and Wellcome Trust Imperial College Centre for Global Health Research, London, UK; Innovation for Health and Development (IFHAD), Laboratory of Research and Development, Universidad Peruana Cayetano Heredia, Lima, Peru; Innovación Por la Salud Y Desarrollo (IPSYD), Asociación Benéfica PRISMA, Lima, Peru

## Abstract

**Background:**

Contacts of tuberculosis index cases are at increased risk of developing tuberculosis. Screening, preventive therapy, and surveillance for tuberculosis are underused interventions in contacts, particularly adults. We developed a score to predict risk of tuberculosis in adult contacts of tuberculosis index cases.

**Methods:**

In 2002–06, we recruited contacts aged 15 years or older of index cases with pulmonary tuberculosis who lived in desert shanty towns in Ventanilla, Peru. We followed up contacts for tuberculosis until February, 2016. We used a Cox proportional hazards model to identify index case, contact, and household risk factors for tuberculosis from which to derive a score and classify contacts as low, medium, or high risk. We validated the score in an urban community recruited in Callao, Peru, in 2014–15.

**Findings:**

In the derivation cohort, we identified 2017 contacts of 715 index cases, and median follow-up was 10·7 years (IQR 9·5–11·8). 178 (9%) of 2017 contacts developed tuberculosis during 19 147 person-years of follow-up (incidence 0·93 per 100 person-years, 95% CI 0·80–1·08). Risk factors for tuberculosis were body-mass index, previous tuberculosis, age, sustained exposure to the index case, the index case being in a male patient, lower community household socioeconomic position, indoor air pollution, previous tuberculosis among household members, and living in a household with a low number of windows per room. The 10-year risks of tuberculosis in the low-risk, medium-risk, and high-risk groups were, respectively, 2·8% (95% CI 1·7–4·4), 6·2% (4·8–8·1), and 20·6% (17·3–24·4). The 535 (27%) contacts classified as high risk accounted for 60% of the tuberculosis identified during follow-up. The score predicted tuberculosis independently of tuberculin skin test and index-case drug sensitivity results. In the external validation cohort, 65 (3%) of 1910 contacts developed tuberculosis during 3771 person-years of follow-up (incidence 1·7 per 100 person-years, 95% CI 1·4–2·2). The 2·5-year risks of tuberculosis in the low-risk, medium-risk, and high-risk groups were, respectively, 1·4% (95% CI 0·7–2·8), 3·9% (2·5–5·9), and 8·6%· (5·9–12·6).

**Interpretation:**

Our externally validated risk score could predict and stratify 10-year risk of developing tuberculosis in adult contacts, and could be used to prioritise tuberculosis control interventions for people most likely to benefit.

**Funding:**

Wellcome Trust, Department for International Development Civil Society Challenge Fund, Joint Global Health Trials consortium, Bill & Melinda Gates Foundation, Imperial College National Institutes of Health Research Biomedical Research Centre, Foundation for Innovative New Diagnostics, Sir Halley Stewart Trust, WHO, TB REACH, and Innovation for Health and Development.

## Introduction

Contacts of people with tuberculosis are at high risk of also developing tuberculosis.^1^ In low-income and middle-income countries, WHO recommends investigation of contacts in all households of index cases with pulmonary tuberculosis. Investigation should involve screening and surveillance, with the aim of promptly diagnosing tuberculosis and providing preventive therapy for contacts judged to be at the highest risk.^2^ However, resources for contact investigation are often limited in national tuberculosis programmes, and adult contacts are rarely prioritised and frequently do not complete screening or take preventive therapy.^3^ Furthermore, the tests for latent tuberculosis infection that are commonly used to guide prescription of preventive therapy, such as the tuberculin skin test (TST) and interferon-γ-release assays, are technically and logistically complicated.^4,5^ These factors lead to difficulties in assessing who has latent tuberculosis or, when infection is confirmed, who is at high risk of progression to disease and, therefore, most likely to benefit from preventive therapy.

International guidelines generally recommend that only contacts younger than 5 years or those who have HIV infection should receive preventive therapy. To end the tuberculosis epidemic, however, there is widespread recognition that use of preventive therapy needs to be scaled up and to target more effectively people at high risk of developing tuberculosis.^4,6^ Contacts of patients with tuberculosis comprise one such group, but have heterogeneous risk, making effective and targeted use of preventive therapy challenging.

Several factors associated with index cases, households, and contacts are established for risk of latent tuberculosis and progression to disease among contacts.^7^ In this study we aimed to use these factors to derive a score to predict the risk of tuberculosis in adult contacts. Due to the challenges of the TST, we designed a score that could be used without testing for latent tuberculosis infection. Subesequently, we aimed to validate the risk score externally in an independent population.

## Methods

### Study design and participants

We did a prospective derivation study in Peru that used data from contacts of index cases of pulmonary tuberculosis in Ventanilla, an area characterised by desert shanty towns in the northern, coastal extension of Lima. This study was followed by an external validation study done in the urban coastal district of Callao, Lima. In both cohorts, index cases were patients registered with government-run health posts who had laboratory-confirmed pulmonary tuberculosis. Contacts were individuals aged 15 years or older who had been in the same house as an index case for more than 6 h/week in the 2 weeks before tuberculosis was diagnosed in the index case. Ethics approval was obtained from the Callao Ministry of Health, Lima, Asociación Benéfica PRISMA, Lima, and Imperial College London, London, UK.

### Derivation cohort

**Figure 1:**
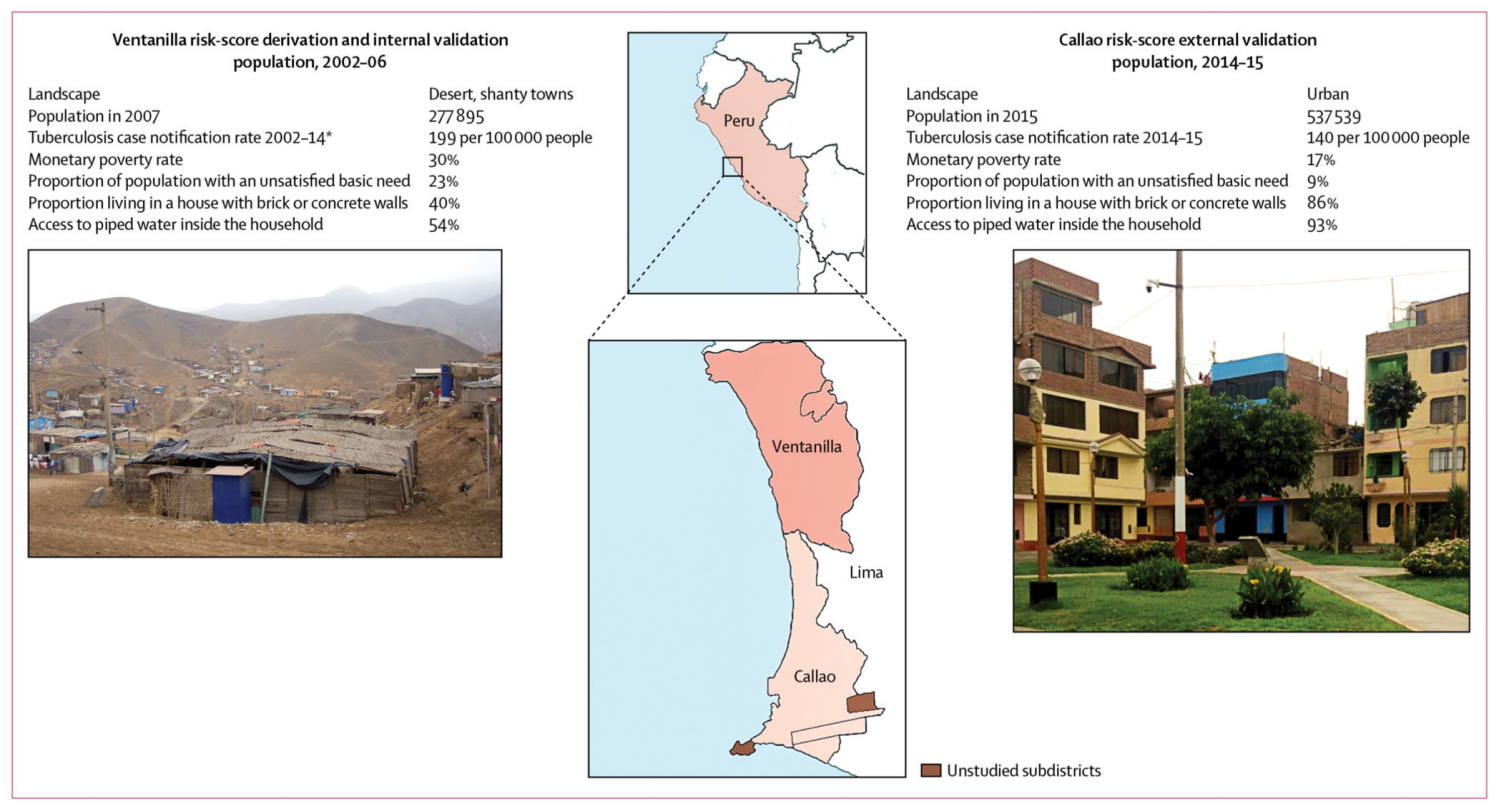
Characteristics of populations and study areas for the derivation and internal validation cohort and the external validation cohort Statistics are reported by the Peruvian Instituto Nacional de Estadistica e Informatica unless indicated otherwise. *Collected collaboratively from government-run health posts.

In 2002–06, we recruited participants from the 15 shantytowns that comprise the Ventanilla district (figure 1). Ventanilla is an area of marked poverty, with many people living in wooden or adobe housing with poor access to essential services and utilities. We invited contacts of index cases to complete a baseline nurse assessment of tuberculosis risk factors (table 1, appendix p 1). Enrolled contacts were followed up until February, 2016, for tuberculosis. For the first 6 months of follow-up we visited households every 2 weeks, during which we offered free sputum smear or culture testing for contacts with tuberculosis symptoms. Thereafter we did household prevalence surveys roughly every 4 years, during which testing was also offered to all contacts. We also asked contacts to self-report tuberculosis diagnosed outside the study area. We ascertained tuberculosis diagnosed within the study area from health-post treatment registers.

### Risk score derivation

For each year of follow-up, we calculated tuberculosis incidence per 100 person-years. We used Cox regression modelling to investigate factors associated with developing tuberculosis and derive a continuous, integer-point risk score from the exact regression coefficients of the final model. We also assessed multiple interactions terms (table 2). We used whole numbers rather than exact regression coefficients to create an easily calculable score for field use. We arbitrarily defined low-risk, medium-risk, and high-risk groups and calculated each contact's 10-year predicted tuberculosis risk with the the exact regression coefficients combined with the baseline survival function (appendix p 2).^8^ To assess the calibration of our integer score to a model that used exact regression coefficients, we derived the 10-year observed risk in risk groups and population deciles of the risk score from Kaplan-Meier functions and compared these with the mean 10-year predicted risks in each group or population decile. To assess the added value of including TST results in our score, we derived a multivariable model that included TST results and compared it with our final model.

### Risk score assessment and internal validation

We calculated the incidence for each risk group, plotted incidence trends, and calculated incidence rate ratios (IRRs) and 95% CIs. Kaplan-Meier functions were plotted to illustrate differences between risk groups in developing tuberculosis after 1, 2·5, 5, and 10 years, and were compared with the log-rank test (appendix p 3). We calculated Harrell's c statistic to assess overall prediction of the continuous risk score. To validate the score internally, we repeatedly fitted the model with 200 bootstrap samples and calculated the optimism-adjusted c statistic (appendix p 3). We calculated the number of contacts needed to treat with preventive therapy in each risk group to prevent one case of tuberculosis over 5 years and 10 years, assuming that preventive therapy was 75% effective on an intention-to-treat basis.^9–11^ Finally, because tuberculosis diagnosed in contacts within 6 months of the index case being diagnosed might not be preventable, we did a sensitivity analysis to assess the risk score excluding contacts who had started tuberculosis treatment within this time frame. All analyses were done with STATA (version 13) and all p values were two-sided.

### External validation

The external validation was done in 17 urban communities in Callao, among participants recruited in 2014–15. This district has marked differences in population demographics, monetary poverty, and material living conditions from Ventanilla (figure 1).^12^ Index cases completed baseline nurse assessments in health posts to provide information on themselves, their households, and their contacts. We used these data to calculate a risk score for each contact (appendix p 3). Contacts were followed up with use of the health-post treatment registers to obtain information on tuberculosis diagnoses until March 1, 2017. We used the same statistical methods as in the internal validation to validate the risk score externally.

### Role of the funding source

The funders had no role in study design, data collection, data analysis, data interpretation, or writing of the report. The corresponding author had full access to all the data in the study and had final responsibility for the decision to submit for publication.

## Results

**Figure 2:**
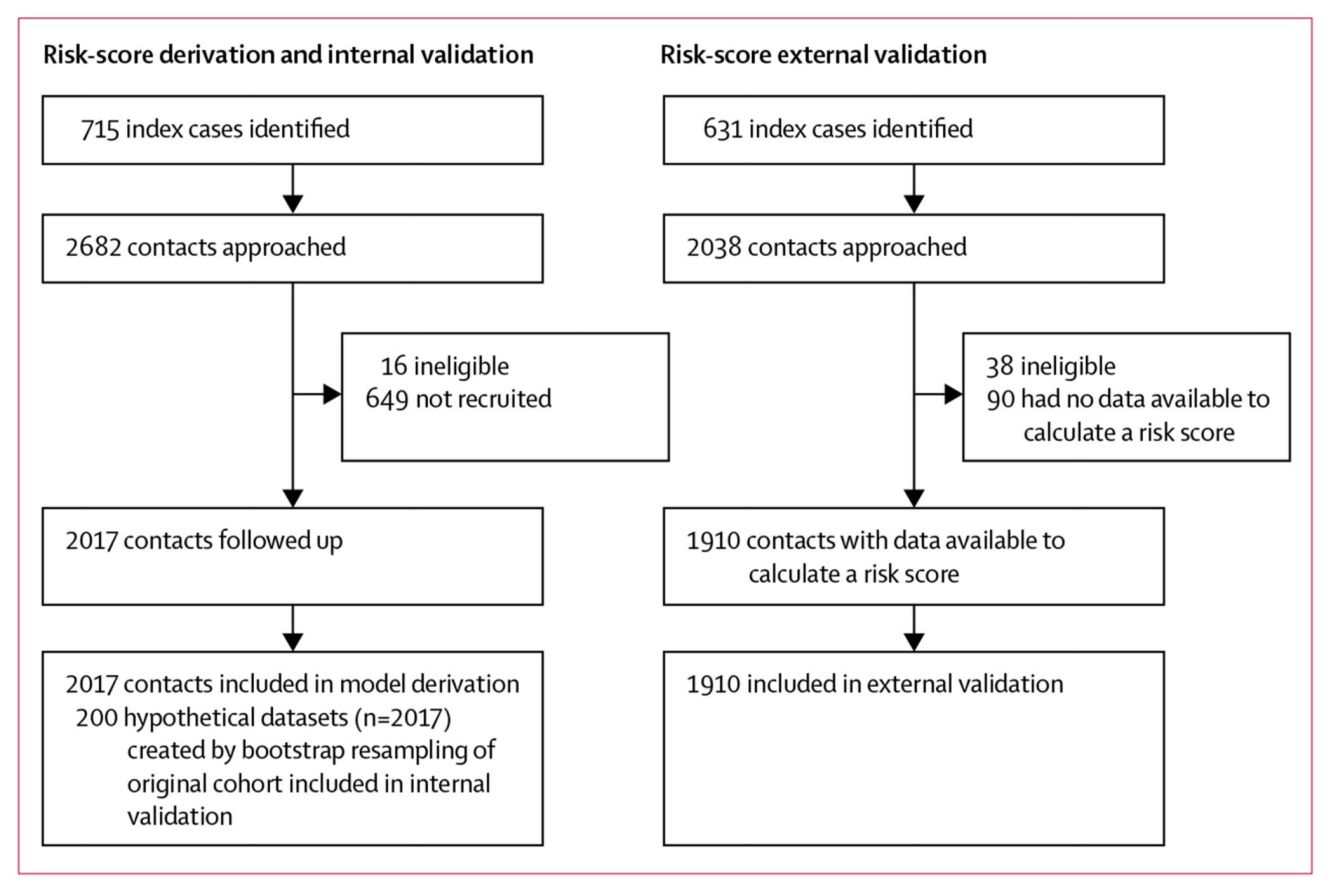
Study profile

Of 2682 contacts approached for inclusion in the derivation cohort, we recruited 2017 (75%, figure 2, table 1, appendix pp 4–6). The median age was 30 years and 40% of contacts were men (table 1). Among those not recruited, the median age was 28 years (IQR 21-42) and 65% were men (95% CI 61-69). Contacts were followed up for a median of 10·7 years (IQR 9·5-11·8).

178 (9%) of 2017 contacts developed tuberculosis during 19 147 person-years of follow-up, giving an incidence of 0·93 per 100 person-years (95% CI 0·80-1·08). Of these, 161 (90%) had microbiologically confirmed disease, were registered in health-post treatment registers, or both, and 17 were treated outside the health posts. The incidence was highest in the first 4 years after exposure, and was more than double the incidence in the local population for the duration of follow-up (appendix p 7).

Contacts who developed tuberculosis were independently more likely to have a low body-mass index, have previously had tuberculosis, be in a high-risk age group, have had sustained exposure to the index case, have had exposure to a male index case, be from a poorer household, have had exposure to indoor air pollution, have a household member with a history of tuberculosis, and live in a household with fewer windows per room than those who did not develop tuberculosis (table 2, appendix pp 4–6). We found no significant interactions between variables but did note some associations between household characteristics (table 2).

**Figure 3:**
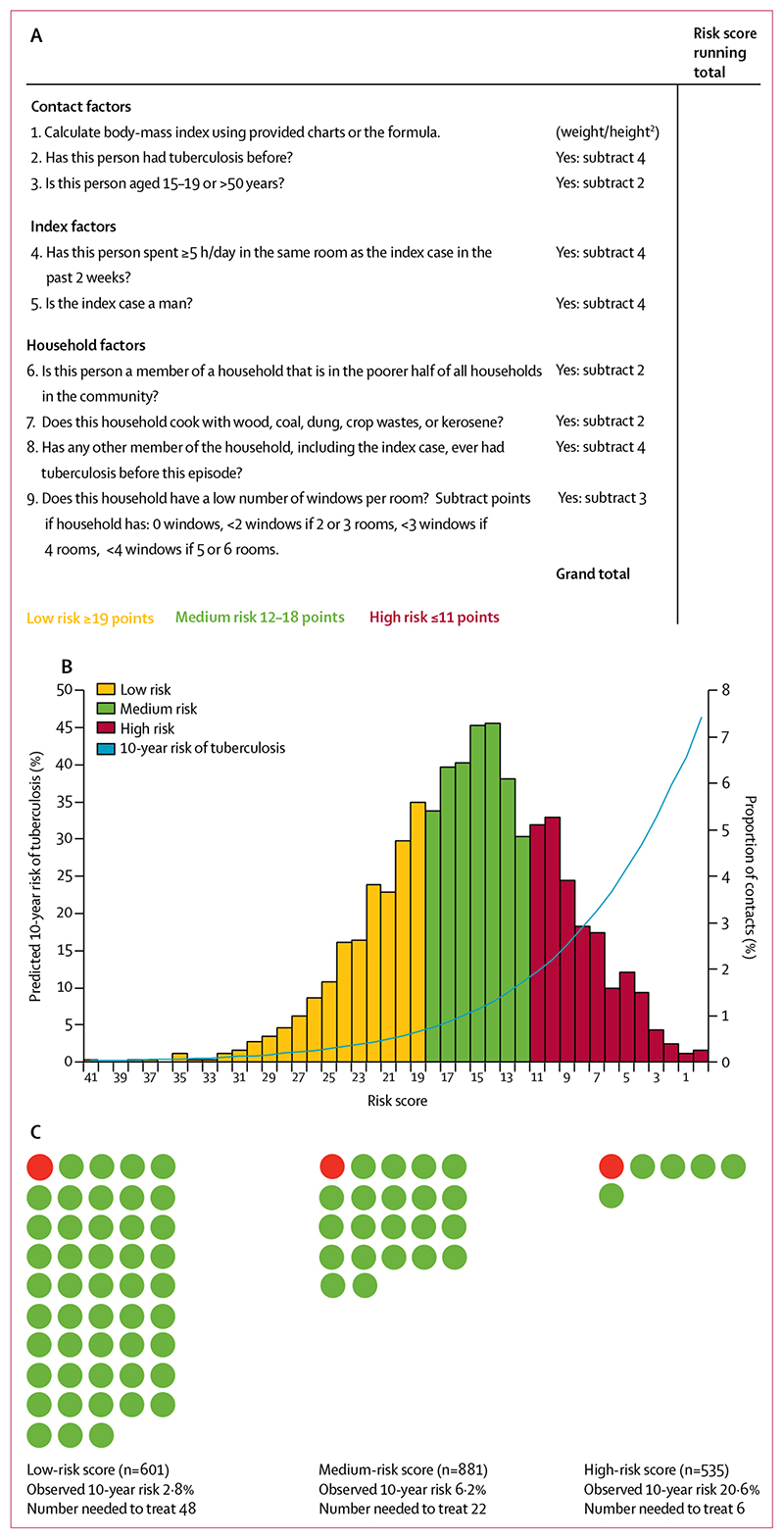
A score to predict risk of tuberculosis in adult contacts of index cases (A) An example risk score for field use. (B) Predicted 10-year risk of tuberculosis plotted against risk scores. (C) Numbers needed to treat with preventive therapy to prevent one case of tuberculosis among contacts, by risk group.

When the regression coefficients and number of integer points assigned to each variable were set (table 2), the score ranges for low, medium, and high risk were set at 19 points or more, 12–18 points, and 11 points or fewer, respectively. An example risk score form is shown in figure 3. Therefore, 601 (30%) contacts were assigned as low risk, 881 (44%) as medium risk, and 535 (27%) as high risk (figure 3). Of the 178 contacts who developed tuberculosis, 17 (10%) were in the low-risk group, 54 (30%) in the medium-risk group, and 107 (60%) in the high-risk group. The 10-year observed risk in the risk groups and population deciles was similar to that predicted with exact regression coefficients (appendix p 8).

**Figure 4:**
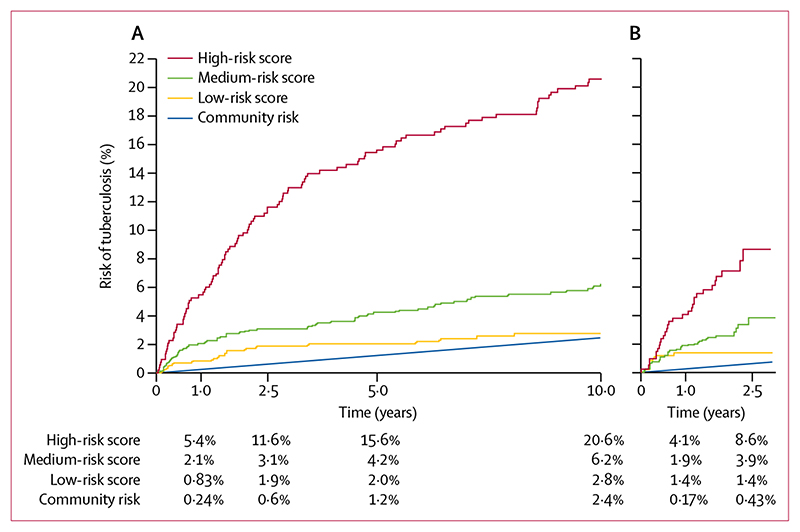
Cumulative observed risk of tuberculosis among contacts, stratified by risk group (A) Ventanilla derivation cohort (n=2017). (B) Callao validation cohort (n=1910). Data are derived from Kaplan-Meier functions. Community risk was defined by the average tuberculosis case notification rate during corresponding years, corrected by 20% to assume under-reporting of cases treated outside the public system, as is the local practice.

Trends in the incidence of tuberculosis for each risk group are shown in the appendix (p 7). The IRR for the high-risk group versus the low-risk group was 8·1 (95% CI 4·8–14, p<0·0001), for the high-risk group versus the medium-risk group was 3·6 (2·6–5·1, p<0·0001), and for the medium-risk group versus the low-risk group was 2·2 (1·3–4·1, p=0·003). The 10-year observed risks in the low-risk, medium-risk, and high-risk groups were 2·8% (95% CI 1·7–4·4), 6·2% (4·8–8·1), and 20·6% (17·3–24·4), respectively (log-rank p<0·0001; figure 4, appendix p 9). The c statistic was 0·72, and after bootstrap resampling internal validation the optimism-adjusted c statistic was 0·71. The risk score predicted risk similarly for contacts of index cases with drug-sensitive tuberculosis (c statistic 0·71) and tuberculosis resistant to isoniazid, rifampicin, or both (c statistic 0·73). In the sensitivity analysis that excluded contacts diagnosed as having tuberculosis within 6 months of the index case being diagnosed, the 10-year observed risks in the low-risk, medium-risk, and high-risk groups were, respectively, 2·1% (95% CI 1·2–3·7), 4·8% (3·5–6·7), and 17·8% (14·7–21·6; c statistic 0·74, log-rank p<0·0001; appendix p 10).

With the assumption of 75% effectiveness of preventive therapy,^9–11^ the numbers needed to treat to prevent one case of tuberculosis over 10 years in the low-risk, medium-risk, and high-risk groups were 48, 22, and six, respectively (figure 3). To prevent one case over 5 years, the corresponding numbers needed to treat were 67, 32, and eight.

**Figure 5:**
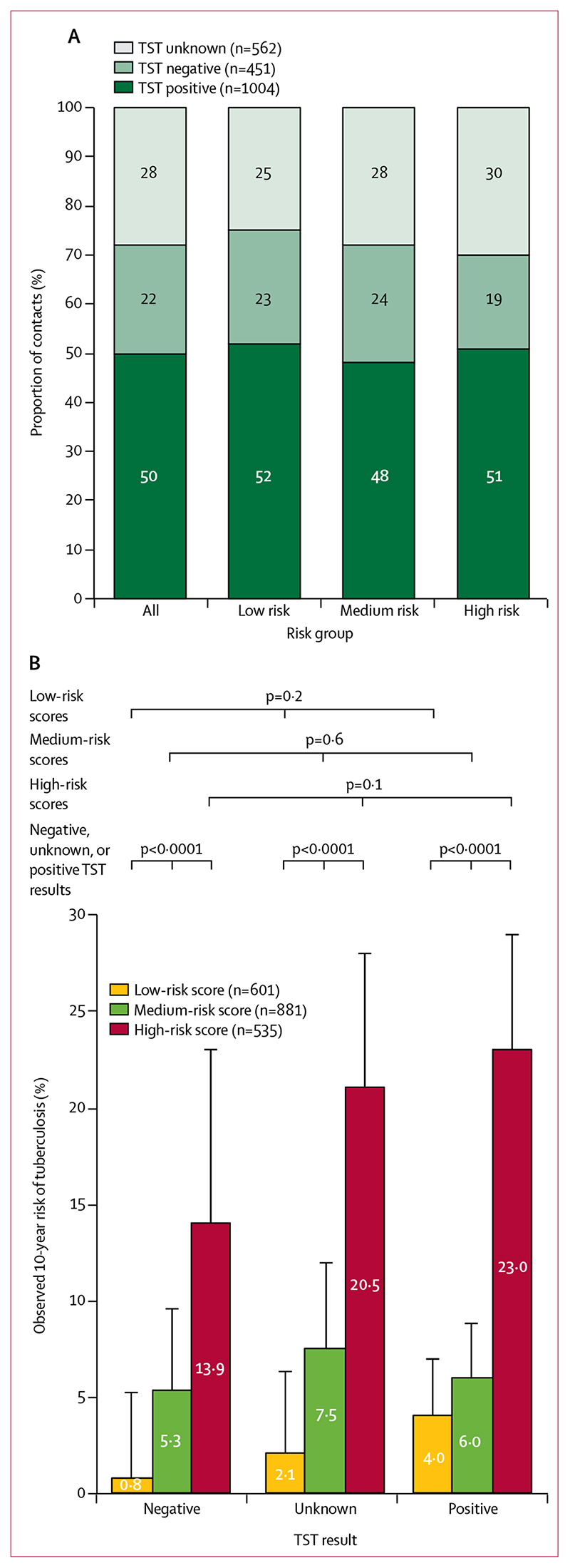
TST results and 10-year observed risk of tuberculosis in the Ventanilla derivation cohort (A) TST results among contacts, stratified by risk group. (B) Observed 10-year risk of tuberculosis stratified by TST results and risk group. Tuberculosis risk was not significantly different within risk groups when stratified by TST result (negative, unknown, or positive), but was significantly different between risk groups for each TST result. Error bars represent 95% CIs. Data are derived from Kaplan-Meier functions. The p values represent log-rank tests for equality of survival functions. TST=tuberculin skin test.

The proportions of contacts who had positive TST results did not differ significantly between risk groups (p=0·13, figure 5). Within each group, there were no significant differences in tuberculosis risk when stratified by TST result (figure 5). Within each TST result (negative, positive, or unknown), our risk score stratified contacts with significantly different tuberculosis risks (log-rank p<0·0001 for each TST result). In a multivariable model including TST results, compared with unknown results, no increased risk of developing tuberculosis was seen for contacts with negative results (adjusted hazard ratio 0·64, p=0·1) or positive results (1·1, p=0·5). As expected, however, TST-positive contacts were more likely to develop tuberculosis than TST-negative contacts (1·8, p=0·02; appendix p 11). Including TST results added little predictive value to our risk model (c statistic 0·73 *vs* 0·72 for the original model).

For the external validation, we recruited 631 index cases and identified 2000 contacts aged 15 years or older. Of these, 1910 (96%) had data available to calculate risk scores. Contacts were followed up for a median 2·0 years (IQR 1·6-2·4). The characteristics of these contacts differed significantly from those in the derivation cohort, particularly for material living conditions (table 1).

Overall, 65 (3%) of 1910 contacts developed tuberculosis during 3771 person-years of follow-up, giving an overall incidence of 1·7 per 100 person-years (95% CI 1·4-2·2). 575 (30%) were classified as low risk, 918 (48%) as medium risk, and 417 (22%) as high risk. The observed risks of tuberculosis at 2·5 years for these risk groups were 1·4% (95% CI 0·70-2·8), 3·9% (2·5-5·9), and 8·6% (5·9-12·6), respectively, and the c statistic was 0·67 (log-rank p<0·0001; figure 4, appendix p 9). In a sensitivity analysis excluding cases diagnosed within 6 months of the index case being diagnosed, the 2·5-year observed risks in the low-risk, medium-risk, and high-riskgroups were 0·18% (95% CI 0·02-1·2), 2·8% (1·6-4·8), and 6·4% (4·0-10), respectively, with a c statistic of 0·75 (log-rank p<0·0001, appendix p 10). Our risk score generally performed well compared with the predicted risk derived from exact regression coefficients, although among contacts with higher scores, the observed 2·5-year risk was marginally lower than the predicted risk (appendix p 12).

## Discussion

In this study of adult contacts of pulmonary tuberculosis index cases from two independent cohorts in Peru, we derived and externally validated a risk score that effectively stratified contacts with significantly different risks of developing tuberculosis. Our score uses data on nine clinical and demographic factors that can be readily collected and that predicted tuberculosis risk independently of TST results. This simple integer-point risk score, which facilitates implementation in the field, yielded results with similar accuracy to those derived from exact regression coefficients. Therefore, we were able to predict risk of developing tuberculosis for at least 10 years after exposure without any laboratory or invasive testing. Use of this risk score could allow a paradigm shift from the current approach of one size fits all to individual-level contact investigation, and might facilitate targeted screening, surveillance, and preventive therapy for adult contacts who are most likely to benefit. This approach could furthermore reduce the number needed to treat with preventive therapy and potentially improve the effects of these interventions, especially in resource-constrained settings.

Preventive therapy provided after tuberculosis exposure and as part of a comprehensive HIV care package is a core component of the WHO End TB Strategy.^6^ It provides robust and sustained protection, especially to individuals without HIV, not only to the recipient but also to individuals at risk of secondary transmission.^4^ In Alaska, USA, where tuberculosis was endemic in the 1950s, the effects of preventive therapy were sustained for at least 20 years.^13^ In Brazil, the THRio study showed that 6 months of preventive therapy had 83% efficacy in HIV-infected individuals for at least 7 years.^14^ In a setting such as Peru, which has medium tuberculosis incidence and low HIV prevalence and is epidemiologically similar to Alaska in the 1950s, preventive therapy after exposure has the potential to confer long-term protection to contacts at high risk of tuberculosis, and is likely to become a priority for the national tuberculosis programme as control efforts focus increasingly on prevention. Of note, our score includes adults whose risk of hepatitis related to preventive therapy increases with age.^10^ Implementing our score should improve the risk-to-benefit ratio and allow the risks, costs, and inconveniences to be better informed and, hence, restricted to the recipients who will benefit most.

Our risk groups were arbitrarily defined for this study. In practice, the risk score could be used with different cutoffs depending on contact and prescriber preferences and the availability of resources. Given the high overall risk of tuberculosis in the two cohorts we assessed, our findings might also support the conclusion that preventive therapy should be given to all contacts, but with prioritisation of those in the high-risk group. Furthermore, the differences in early tuberculosis incidence between risk groups (appendix p 7) highlight a role for our score as an adjunct to prioritise screening, educational interventions, and future surveillance for contacts at highest risk. In a previous study, a similar algorithm was derived to predict risk of tuberculosis among child contacts, but relied on TST results and was hindered by the uncertainty of diagnosing tuberculosis in children.^15^ Another study described a simple algorithm that incorporated exposure variables into a score to predict latent tuberculosis among children younger than 15 years, which might facilitate targeted preventive therapy.^16^ A further study derived and validated a blood RNA signature for predicting tuberculosis among adolescents and adults.^17^ Although the results were promising, the gene signature only had predictive ability for up to 18 months, and the technology and infrastructure required are unlikely to be feasible for implementation in resource-constrained settings. By contrast, our risk score predicted risk of developing tuberculosis with similar accuracy but for at least 10 years after exposure.

In our derivation cohort, positive TST results were common and had similar frequency in all three risk groups. In high-incidence settings, TST has reduced specificity and limited power as a predictor of tuberculosis.^18^ Furthermore, TST might show falsenegative results in people at the highest risk of developing tuberculosis, such as those who are undernourished or have HIV, and false-positive results in people who have received BCG vaccinations.^19,20^ We chose not to include TST results in our model because these limitations are exacerbated by operational barriers, including needing trained staff to do the test, repeated clinic visits, and reduced availability in resource-constrained settings.^3,5^ We believe this approach is justified because the addition of TST results to the model did not significantly improve its predictive power. Importantly, our score accurately stratified contacts with differing risks of tuberculosis independently of TST results.

The variables that make up our score include several established risk factors for developing tuberculosis and highlight the complex relations between tuberculosis risk and characteristics of contacts, index cases, and households.^7^ Our results corroborate findings from a meta-analysis that showed a consistent log-linear relation between body-mass index and tuberculosis incidence.^21^ Sustained exposure to the index case and the contact having previous tuberculosis are both well established as risk factors for developing tuberculosis, particularly in child contacts.^16^ We found associations between risk of developing tuberculosis and age being 15–19 years or 50 years or older. These results are similar to the national and regional data in Peru on tuberculosis incidence.^22^ Operationally, users of our score are required to determine whether a contact is from the poorer half of households in the community. Although socioeconomic position will vary between settings, users could be assisted in making this assessment by using a setting-specific poverty index similar to the ones we used. Moreover, as WHO places increasing emphasis on documenting tuberculosis-related costs, the process of defining socioeconomic position is likely to become more clearly defined.^23^ Our study adds to the evidence suggesting that exposure to indoor air pollution and living in poorly ventilated households increase the risk of developing tuberculosis.^24,25^ A strength of our assessment is that we did not use expensive equipment to quantify exposure to particulates or ventilation and, therefore, our operational definitions reflect real-world data that a tuberculosis programme could realistically collect. On a broader level, government departments might target these risk factors directly through providing clean cooking stoves and fuel, education on maintaining ventilation, and, ultimately, improved housing to communities with a high tuberculosis burden.

Global tuberculosis control efforts aim to improve the diagnosis and prevention of all cases of tuberculosis, irrespective of the primary source of infection. Therefore, we did not use molecular techniques to identify tuberculosis strains and confirm transmission from index cases to contacts because we believed it would not affect our conclusions. Although we did not confirm transmission, we found that index case smearpositivity grade, and self-reported cough frequency did not predict risk of tuberculosis in contacts (appendix pp 4–6). Our findings suggest that these measures are unreliable markers of infectiousness and support the use of objective acoustic parameters, such as cough monitors, viability microscopy, and cough aerosol cultures, to assess infectiousness in future research.^26,27^ In contrast to previously published data,^28^ we found no evidence that contacts of index cases of multidrugresistant tuberculosis were at lower risk than contacts of index cases of drug-sensitive tuberculosis. Furthermore, the high burden of tuberculosis seen among contacts of index cases with multidrug-resistant tuberculosis highlights the importance of long-term surveillance and use of available preventive therapy to reduce ongoing community transmission.

Other strengths of this study include comprehensive follow-up to ascertain diagnosis of tuberculosis, robust internal validation, and subsequent external validation in a distinct population with different tuberculosis epidemiology at a different time. Importantly, the risk factors included in our model have documented associations with tuberculosis across diverse locations, including high-income countries,^29,30^ which suggests that the score will be adaptable across a broad range of settings. Limitations of our study include a risk of selection bias in the derivation cohort, although the likelihood of bias was reduced by most contacts being followed up and subsequent high numbers of contacts being recruited in the external validation cohort. We were unable to assess how risk factors changed with time and did not collect data from contacts on subsequent tuberculosis exposures. However, these data were not desirable for our objectives as we aimed to derive a tool specifically for use during initial contact investigation. Furthermore, we did not perfectly characterise all established risk factors for developing tuberculosis and could detect no association with comorbidities and tuberculosis, perhaps because self-reporting comorbidities might have underestimated their prevalence. Although our HIV data were limited due to universal testing not being available, population prevalence was low^31^ and was unlikely to have significantly affected our results. Importantly, HIV-infected individuals and those with diabetes are already prioritised for interventions and, therefore, our score does not discriminate against these people but acts as an adjunct to identify others at high risk. Finally, the data used in our external validation cohort were reported by the index case, including estimates of contact weight and height, which reflects the operational data that might be collected by health-care staff during routine contact investigation. We did not have data available on windows per room, and our variable characterising sustained exposure to the index case was defined differently from that used in the derivation cohort. Despite these differences, the score had good discrimination under operational conditions in the external validation cohort, whether including or excluding contacts diagnosed as having tuberculosis within 6 months of the index case being diagnosed. Although the observed risk among contacts in the high-risk group was slightly lower in the validation cohort than the predicted risk, this difference might have arisen because ascertainment only included cases identified through health-post treatment registers without household visits. Overall, however, our derived and externally validated risk score offers an intuitive and practical tool for initial contact investigations to stratify adult contacts and identify those at the highest risk of developing tuberculosis.

## Supplementary Material

See Online for appendix

Supplementary file

## Figures and Tables

**Table 1:** Characteristics of the derivation and external validation cohorts

	Ventanilla derivation cohort (n=2017)	Callao external-validation cohort (n=1910)	p value
**Contacts**			
Age at recruitment (years)	30 (22–43)	38 (25–52)	<0.0001*
High-risk age group (15–19 or >50 years)	602 (30%)	761(40%)	<0.0001
Men/women	814 (40%)/1203 (60%)	914 (48%)/996 (52%)	<0.0001
Body-mass index (kg/m^2^)†	25.2 (4.2)	25.6 (3.9)	0.005‡
History of tuberculosis	222 (11%)	197 (10%)	0.5
**Index cases**			
Age at recruitment (years)	26 (20–36)	28 (21–42)	<0.0001*
Men/women	1210 (60%)/807 (40%)	1240 (65%)/670 (35%)	0.001
Sputum smear status			<0.0001
Negative	53 (3%)	126 (7%)	
1+	726 (36%)	1104 (58%)	
2+	635 (31%)	402 (21%)	
3+	603 (30%)	278 (15%)	
Drug sensitivity			0.004
Sensitive	1620 (80%)	1498 (78%)	
Resistant to isoniazid	171 (9%)	223 (12%)	
Multidrug resistant§	226 (11%)	189 (10%)	
Sustained exposure to index case¶	1071 (53%)	1188 (62%)	<0.0001
Migrant from coastal, mountainous, or jungle area of Peru	1054 (52%)	414 (22%)	<0.0001
**Households**			
Exposure to indoor air pollution||	698 (35%)	20 (1%)	<0.0001
Any household member with a history of tuberculosis	742 (37%)	801 (42%)	0.001
Fewer windows per room**	797 (40%)		
Wall material			<0.0001
Adobe	223 (12%)	75 (4%)	
Wood	842 (42%)	257 (13%)	
Cement or brick	942 (47%)	1578 (83%)	
Floor material			<0.0001
Dirt	641 (32%)	105 (6%)	
Cement	1212 (60%)	1301 (68%)	
Tiles or laminated surface	164 (8%)	504 (26%)	
Access to piped water inside the house	1049 (52%)	1852 (97%)	<0.0001
Access to a toilet inside the house	972 (48%)	1851(97%)	<0.0001
Electric lighting	1911 (95%)	1868 (98%)	<0.0001
Asset ownership			<0.0001
Television	1859 (92%)	1865 (98%)	
Stove	1979 (98%)	1843 (96%)	0.002
Fridge	988 (49%)	1604(84%)	<0.0001
Head of household did not complete secondary education	1155 (57%)	730 (38%)	<0.0001

Data are median (IQR), number (%), or mean (SD). All p values stated represent χ^2^ tests unless otherwise stated. See appendix pp 1–6 for a full description of these variables and the rationale for their definition.

* Mann-Whitney *U* test.

† Adjusted with WHO BMI-for-age charts (for ages 15, 16, 17, and 18 years, multiplied by 1·12, 1·09, 1·05, and 1·02, respectively).

‡ Two sample *t* test.

§ Defined by initial prescription of a multidrug-resistant tuberculosis regimen or by microbiological evidence of resistance to rifampicin and isoniazid.

¶ ≥5 h/day to index case in the 2 weeks before index case diagnosis (derivation cohort) or ≥60 h while index case had cough (validation cohort). ||Living in a household that cooked predominantly with kerosene (or occasionally solid fuels: wood, coal, animal dung, or crop wastes).

** Defined as <0.67 windows per room in the derivation cohort; data not available for validation cohort and, therefore, to calculate a risk score for contacts we gave all participants an average value (appendix pp 1–3).

**Table 2:** Multivariable Cox regression analysis of factors associated with tuberculosis in the derivation cohort

	Unadjusted hazard ratio (95%CI)	Adjusted hazard ratio (95%CI)	p value	Regression coefficient	Points assigned in risk score*
**Contacts**					
BMI	0.87 (0.84–0.91)	0.87 (0.83–0.91)	<0.0001	–0.138	Value of BMI
History of previous tuberculosis	2.0 (1.4–2.9)	1.8 (1.2–2.6)	0.005	0.566	−4
High-risk age group (15–19 or >50 years)	1.5 (1.1–2.0)	1.3 (0.96–1.8)	0.09	0.272	−2
**Index cases**					
Sustained exposure to index case	1.6 (1.2–2.2)	1.8 (1.3–2.4)	0.0003	0.573	−4
Exposure to male index case	1.5 (1.0–2.1)	1.7 (1.2–2.4)	0.001	0.554	−4
**Households**					
Lower community household socioeconomic position†	1.4 (1.0–2.0)	1.3 (0.95–1.8)	0.1	0.281	−2
Exposed to indoor air pollution	1.7 (1.3–2.4)	1.4 (0.97–1.9)	0.07	0.302	−2
Any household member with a history of tuberculosis	1.7 (1.2–2.3)	1.7 (1.2–2.3)	0.001	0.530	−4
Fewer windows per room	1.6 (1.1–2.2)	1.6 (1.2–2.2)	0.004	0.469	−3

The test of proportional hazards assumption for the entire model was χ^2^=7·27, p=0·61. BMI=body-mass index.

* Calculated by multiplying the Cox regression coefficient by a constant (–7·25) and rounding to the nearest integer; the constant was chosen so that the exact BMI value could be used in the score, using the reciprocal (1/–0·138).

† Measured with a household poverty index that combined 12 variables characterising education, access to services and material living conditions into a continuous variable that was dichotomised into two equal categories. The following interactions showed no significant associations and made no significant differences to the model when tested using the likelihood-ratio test, and, therefore, were excluded from the final model: sustained exposure and sex of index case (p_interaction_=0·8); sex of index case and index case smear positivity status (p_interaction_=0·7); exposure to indoor air pollution and fewer windows per room (p_interaction_=0·5); sustained exposure and fewer windows per room (p_interaction_=0·6); and sex of index case and socioeconomic position (p_interaction_=0·07). Among the household characteristics, the only significant associations were between exposure to indoor air pollution and socioeconomic position (p<0·0001); exposure to indoor air pollution and fewer windows per room (p=0·0003); and indoor air pollution and any household member with a history of tuberculosis (p<0·0001).

## Research in context

### Evidence before this study

We searched PubMed for studies published until April 30, 2017, with the search term “tuberculosis/risk [MesH] OR tuberculosis/prevention [MesH] OR tuberculosis/prediction [MesH] OR tuberculosis/score”. We found several reviews of tuberculosis contact investigation and preventive therapy that highlighted the low attention given to such interventions worldwide and emphasised the urgent need for scale-up if the objectives of WHO’s End TB Strategy are to be achieved. The low attention is particularly true for adults without HIV infection, who in our study setting and worldwide account for most tuberculosis disease among contacts. We found many studies and reviews characterising risk factors for tuberculosis, although only one attempted to define a method to predict tuberculosis risk in adults with assessment of a blood RNA signature. Tools that rely on invasive laboratory tests are promising but have limited potential for use in resourceconstrained settings, where much of the world’s tuberculosis occurs.

### Added value of this study

We derived and externally validated a score to predict the risk of tuberculosis in contacts aged 15 years or older of patients with laboratory-confirmed pulmonary tuberculosis. This risk score, which was based on nine clinical and demographic risk factors, accurately stratified contacts with large differences in tuberculosis risk, independently of tuberculin skin test results. Our risk score shows that while well nourished contacts living in well ventilated houses among residents with no history of tuberculosis and low exposure to the index tuberculosis case might have a predicted 10-year risk of one in 100, underweight contacts living in poorly ventilated houses among residents with a history of tuberculosis and substantial exposure to the index tuberculosis case might have a predicted 10-year risk of tuberculosis approaching one in three.

### Implications of all the evidence

Despite advances in tuberculosis diagnosis and treatment, to reduce tuberculosis incidence worldwide, prevention of new cases in high-risk groups will need to be improved. Tuberculosis screening, preventive therapy, and surveillance of contacts are underused interventions. Our risk score could facilitate their scale-up through the identification of high-risk adult contacts of index cases, enabling these interventions to be more effectively targeted to those who are most likely to benefit. Integrated within the Community Randomised Evaluation of a Socioeconomic Intervention to Prevent TB study, we have started assessing whether targeted preventive therapy given based on this risk score, combined with socioeconomic support to empower access to care, can prevent tuberculosis among contacts.
