# Supplementary material for: A score to predict and stratify risk of tuberculosis in adult contacts of tuberculosis index cases: a prospective derivation and external validation cohort study
Source: Lancet Infect Dis. Author manuscript; Available in PMC 2021 Jul 8. (PMC7611139; doi:10.1016/S1473-3099(17)30447-4)
Supplement: Supplementary file [file EMS128337-supplement-Supplementary_file.pdf]

THE LANCET Infectious Diseases

Supplementary webappendix

This webappendix formed part of the original submission and has been peer reviewed. We post it as supplied by the authors.

Supplement to: Saunders MJ, Wingfield T, Tovar MA, et al. A score to predict and stratify risk of tuberculosis in adult contacts of tuberculosis index cases: a prospective derivation and external validation cohort study. *Lancet Infect Dis* 2017; published online August 18. http://dx.doi.org/10.1016/S1473-3099(17)30447-4.

APPENDIX METHODS

Study design

We used data collected from two independent cohorts from the northern, coastal extension of Lima, Peru to first derive a risk score, and then subsequently externally validate it (Figure 1).

Derivation cohort participants

To derive a risk score, we used data from a prospective cohort study embedded within a randomized trial of micronutrient supplementation (vitamin D, vitamin A and zinc) to prevent TB among contacts, with enrolment from October 2002 until June 2006 and follow up until February 2016 (Figure 2). Since micronutrient supplementation was found to not influence TB risk, all contacts were included in the derivation cohort, irrespective of allocation.[1] Participants were recruited from the 15 desert shantytowns comprising Ventanilla district with an estimated population of 277,895 in 2007.[2] Figure 1 demonstrates key statistics reported by the Peruvian Instituto Nacional de Estadistica e Informatica (INEI) for Ventanilla.[3,4] The TB case notification rate collected collaboratively from government-run health posts in these 15 shantytowns averaged 199/100,000 people/year between 2002-2014. National HIV prevalence is low (<0.5% in the general population and <5% among patients with TB).[5] *Index cases* were defined as patients registered with government-run health posts who were diagnosed with laboratory confirmed pulmonary TB, which during this period usually implied sputum smear microscopy positive TB. We deliberately focused recruitment on contacts of patients with smear-positive TB as these contacts are known to have a higher risk than those of smear-negative patients, who were generally treated for presumed TB without bacteriological confirmation in our setting. Multi-drug resistant (MDR) TB was defined in patients initially prescribed an MDR treatment regimen or who had microbiological evidence of resistance to rifampicin and isoniazid. Index cases were eligible if they had at least one contact aged ≥15 years who agreed to participate. If a patient was treated twice during the study, we only invited them to participate the first time. *Contacts* were defined as individuals aged ≥15 years who reported being in the same house as the recruited index case for over 6 hours/week in the two weeks preceding index case diagnosis, independently of where they lived. Our research focused on contacts aged ≥15 years because TB is rarely laboratory confirmed in those aged <15 years, national policy during this period was that contacts aged <15 years should be considered for PT, and consequently few contacts aged ≥15 years were eligible for PT. Contacts were ineligible if they were taking TB treatment at the time of recruitment but were recruited independently of any symptoms they had. We obtained written informed assent and/or consent from all index cases and their contacts.

Derivation cohort measurements

We identified index cases by collaboration with government-run health posts and sought to immediately recruit them and their contacts. Participants completed a questionnaire characterising demographics; medical history including self-reported comorbidities such as HIV infection and previous TB; and other TB risk factors.[6] We offered baseline TST to all contacts but TST conversion was not studied.[7] Height and weight were measured and body mass index (BMI) calculated. BCG vaccination was assessed by examination for scars by a trained nurse, complemented by participant and family recall. Relative community household socioeconomic position was measured using a household poverty index which combined 12 variables characterising education, access to services and material living conditions into one continuous variable that we dichotomised into two equal categories.[6] Exposure to indoor air pollution (IAP) was defined as living in a household that cooked predominantly with kerosene (or occasionally solid fuels: wood, coal, animal dung or crop wastes). All index cases were invited to give a sputum sample, which was tested by smear microscopy and by the microscopic-observation drug-susceptibility (MODS) assay.[8]

After the initial assessment, we visited households every 2-4 weeks for the duration of the index case's treatment. During these visits, we offered free sputum TB smear, culture and drug-sensitivity tests for contacts with symptoms suggestive of TB. In addition, we visited all recruited households approximately every four years after recruitment, asked about TB diagnoses, and offered free sputum testing to all contacts. All samples obtained were processed using smear microscopy and cultured using the

MDR/XDR-TB Colour Test thin-layer agar assay.[9] We considered a contact to have developed TB either by microbiological confirmation in sputum collected during follow-up, ascertainment of a diagnosis of extra-pulmonary or pulmonary TB confirmed in health post treatment registers until 1st February 2016, or self-reported diagnosis outside the study area. Time to TB was defined as the date the index case initiated treatment until the date their contact first initiated treatment, or if this date was unavailable, until the date their contact was diagnosed. For contacts who did not develop TB, follow-up was censored at the date they were last known to be alive and free of TB.

Risk score derivation

Continuous data were summarised by their means and standard deviations (SD) for parametric data and medians and interquartile ranges (IQR) for non-parametric data. Categorical data were summarised as proportions with 95% confidence intervals (95%CI). To facilitate comparison of BMI across all ages, we adjusted BMI for participants aged 15-18 years estimating what their BMI would have been if they were aged 19 years using the WHO BMI-for-age tables.[10] BMI is presented as a continuous variable as the relationship with TB incidence was linear and most strongly influenced risk. To facilitate field implementation of a score, other continuous variables were examined for linearity with TB incidence and were dichotomised if linearly associated. Specifically, prolonged exposure to the index case was defined if the contact spent at least five hours in the same room as the index case in the two weeks preceding diagnosis, and fewer windows per room was defined if households had less than 0.67 windows per room. We examined age-specific incidence in five-yearly categories and defined a dichotomous variable with contacts aged 15-19 and >50 years compared to contacts aged 20-50 years because contacts aged 15-19 and >50 years had a similar TB incidence, which was significantly higher than the incidence in those aged 20-50 years.

For each year following TB exposure, we calculated TB incidence rates (IR) per 100 person-years and generated 95%CI based on the Poisson distribution. For each risk factor (appendix, pages 4-6), we calculated overall IR and generated incidence rate ratios (IRR). Cox-proportional hazards models with robust standard errors adjusted for household clustering using a clustered sandwich estimator were fitted to investigate factors associated with TB. We first performed univariable analyses testing each variable alone, and subsequently included plausible variables that showed an association with $p<0.2$ in a multivariable model. The maximal multivariable model was reduced by eliminating variables sequentially and comparing each model with likelihood ratio tests. We evaluated multiple interaction terms described in the footnote to Table 2. The proportional-hazards assumption was tested by examination of the Schoenfeld residuals. All analyses were performed using STATA (version 13, StataCorp) and all *p*-values were two-sided.

To derive a risk score, we assigned integer points proportional to each variable's regression coefficient from the final model. We used whole numbers rather than exact regression coefficients to create an easily calculable score for field use. A risk score was calculated for each contact by combining these points with each contact's characteristics. Three groups: low-risk (≥19 points); medium-risk (12-18 points); and high-risk (≤11 points) were arbitrarily defined so that the high-risk group predicted approximately 50% of the cohort TB burden, and the high and medium groups together predicted approximately 90%. We estimated each contact's 10-year predicted TB risk by combining the exact regression coefficients from the final model with each contact's characteristics, and with the baseline survival function for all contacts.[11] The baseline survival function was estimated from the Cox regression model based on zero values of centred continuous risk factors with all binary risk factors set to zero, and the 10-year value was extracted. To assess calibration of the integer score compared to the exact model we derived the 10-year observed risk in risk groups/population deciles from Kaplan-Meier functions and compared this with the mean 10-year predicted risk in each risk group/population decile. To assess the added value of including TST results in a score, we generated a further multivariable model including TST results and evaluated its predictive performance.

Risk score evaluation

IR were calculated for each risk group, incidence trends plotted and IRR generated to compare incidence between our defined risk groups. Kaplan-Meier functions were derived, plotted and compared using the log-rank test. We generated histograms illustrating differences between risk groups after 1, 2.5, 5 and 10 years and also compared this to the community rate, which was calculated using the average TB case notification rate during corresponding years, corrected by 20% to assume under-reporting of cases treated outside of the public system, as is the local practice.[12] Harrell's C-statistic was calculated to assess overall prediction of the continuous risk score. The number of contacts needed to treat (NNT) with PT in each risk group to prevent one TB case over 5 and 10 years was calculated assuming PT was 75% effective on an intention to treat basis.[13–15] We further evaluated score performance stratifying by TST result and index drug sensitivity result. As TB diagnosed in contacts in the first 6 months following index case diagnosis may not be preventable, we performed a sensitivity analysis evaluating the score excluding contacts who initiated TB treatment within 6 months of the index case.

Risk score internal validation

Because statistical models are generated to provide the best fit for the available data, they typically provide an optimistic assessment of predictive ability.[16,17] To correct for this optimism and internally validate the score, we repeatedly fit the model with 200 bootstrap samples and calculated the optimism-adjusted C-statistic. This method is recommended for internal validation as it produces the most robust estimates of external performance.[17]

Risk score external validation

To externally validate the score in an independent population, we used data collected as part of our research group's longitudinal cohort study of patients with TB. In 2014 and 2015, we expanded activities to include 17 urban communities in Callao, Bellavista and La Perla districts (referred to subsequently as Callao), which had a combined population of 537,539 in 2015.[4,18–20] The INEI report statistics for Ventanilla separately from urban Callao and consider each a distinct area due to significant differences in population demographics, monetary poverty and material living conditions (Figure 1).[4] Callao is considered to have a higher socioeconomic position than Ventanilla and is the country's principal port.[4] The TB case notification rate collected collaboratively from government-run health posts in these 17 communities averaged 140/100,000 between 2014 and 2015. We identified all index cases commencing TB treatment in these health posts and invited their household to participate using the same definitions as for the derivation cohort. We obtained written informed assent and/or consent from all index cases on the household's behalf.

All index cases completed a questionnaire in health posts exploring index case, household, and contact characteristics. The advantage of this approach is that the data collected are operational and a more realistic estimate of data that might be collected by health personnel at the time of contact investigation. We used these prospectively collected data to retrospectively calculate a risk score for each contact. Data were available on all variables included in the score except for the number of windows/room a house had. For this variable, we assigned all participants a mid-range value. Our data on contact exposure to the index case differed in this cohort because instead of quantifying daily exposure, we asked the total hours spent with the index case whilst the index case had symptoms. As for the derivation cohort, we examined the association between this variable and TB incidence and because the relationship was linear, defined contacts as having prolonged exposure if they had spent at least the median value of 60 hours with the index case. Contacts were followed for TB using health post treatment registers until 1st March 2017. We used the same statistical methods described above to externally validate the score. Prediction was assessed by calculating the C-statistic, deriving and plotting Kaplan-Meier functions and generating histograms illustrating differences between risk groups at 1 and 2.5 years post-exposure. Calibration was assessed by comparing the 2.5-year observed risks in population deciles derived from Kaplan-Meier functions with the mean 2.5-year predicted risk in each decile.

Table S1: Characteristics of the Ventanilla derivation cohort, tuberculosis incidence rates and rate ratios, and univariable Cox-regression analysis for associations with tuberculosis.

	Ventanilla derivation cohort (n=2,017)	Tuberculosis incidence rate if factor present (per 100 person-years) (95% CI)	Tuberculosis incidence rate if factor not present (per 100 person-years) (95% CI)	Tuberculosis incidence rate ratio (95%CI)	Unadjusted hazard ratio for tuberculosis (HR) (95%CI)	p value for HR
**CONTACT CHARACTERISTICS**						
General						
Age at recruitment (median years; IQR)	30 (22-43)	NA				
High risk age group (aged 15-19 or >50 years) (%; 95%CI)	30 (28-32)	1.2 (0.95-1.1)	0.81 (0.68-0.98)	1.5 (1.1-2.0)	1.5 (1.1-2.0)	0.01
Sex (% male; 95%CI)	40 (38-43)	1.0 (0.82-1.3)	0.86 (0.71-1.1)	1.2 (0.87-1.6)	1.2 (0.87-1.6)	0.3
Smoker[1] (≥1 cigarette smoked in the last week; %; 95%CI)	7.7 (6.1-9.6)	0.56 (0.21-1.5)	0.73 (0.57-0.94)	0.76 (0.20-2.1)	0.77 (0.28-2.1)	0.6
Drug user[1] (%; 95%CI)	6.4 (4.9-8.2)	0.90 (0.37-2.2)	0.70 (0.55-0.91)	1.3 (0.40-3.1)	1.2 (0.50-3.1)	0.7
Alcohol use (≥1 days intoxicated in previous month) (%; 95%CI)	19 (18-21)	0.65 (0.44-0.97)	1.0 (0.85-1.2)	0.66 (0.41-1.0)	0.65 (0.42-1.0)	0.06
Incomplete schooling (<secondary completed) (%; 95%CI)	57 (54-59)	1.0 (0.85-1.2)	0.79 (0.62-1.0)	1.3 (0.95-1.8)	1.3 (0.95-1.8)	0.1
Food insecurity (went to bed hungry at least 1 day in the last month) (%; 95%CI)	26 (24-27)	1.2 (0.86-1.5)	0.87 (0.74-1.0)	1.3 (0.92-1.9)	1.2 (0.87-1.8)	0.2
Migrant from coastal, mountainous or jungle area of Peru (%; 95%CI)	55 (53-57)	0.80 (0.65-0.99)	1.1 (0.88-1.3)	0.74 (0.54-1.0)	0.74 (0.55-1.0)	0.05
Prescribed preventive therapy (%; 95%CI)	0 (0-0)	NA				
Self-reported co-morbidities (%; 95%CI)						
Known to have HIV infection[2]	0.20 (0.054-0.51)	NA				
Any of the below self-reported co-morbidities	22 (20-24)	0.99 (0.73-1.3)	0.91 (0.77-1.1)	1.1 (0.75-1.6)	1.1 (0.75-1.5)	0.7
Diabetes	1.1 (0.68-1.6)	1.0 (0.30-4.1)	0.93 (0.80-1.1)	1.1 (0.13-4.1)	1.0 (0.26-4.2)	0.95
Heart disease	3.9 (3.1-4.8)	1.3 (0.67-2.4)	0.92 (0.79-1.1)	1.4 (0.62-2.7)	1.3 (0.69-2.6)	0.4
Cancer	0.84 (0.48-1.3)	NA				
Kidney disease	5.1 (4.1-6.1)	0.73 (0.35-1.5)	0.94 (0.81-1.1)	0.78 (0.31-1.6)	0.77 (0.37-1.6)	0.5
Liver disease	4.3 (3.4-5.2)	1.1 (0.6-2.1)	0.9 (0.80-1.1)	1.2 (0.55-2.4)	1.2 (0.59-2.5)	0.6
Autoimmune disease	1.4 (0.92-2.0)	1.5 (0.60-4.1)	0.9 (0.80-1.1)	1.7 (0.45-4.3)	1.6 (0.64-4.2)	0.3
Respiratory disease other than tuberculosis	4.3 (3.4-5.2)	0.62 (0.26-1.5)	0.94 (0.81-1.1)	0.66 (0.21-1.6)	0.66 (0.27-1.6)	0.4
Neurological disease	1.6 (1.1-2.3)	1.2 (0.45-3.2)	0.93 (0.80-1.1)	1.3 (0.35-3.3)	1.3 (0.48-3.5)	0.6
Anthropometry						
Weight (kg) (mean; SD)	61 (11)	NA				
Height (cm) (mean; SD)	156 (8.4)	NA				
Body mass index (mean; SD) (adjusted for age[3])	25.2 (4.2)	NA		0.90 (0.87-0.93)	0.87 (0.84-0.91)	<0.001
Previous TB exposure						
BCG vaccination scar(s) visible (%: 95%CI): *0*	12 (10-13)	0.87 (0.55-1.4)		*Reference*	*Reference*	*Reference*
1	47 (44-49)	0.97 (0.80-1.2)		1.1 (0.67-1.9)	1.1 (0.70-1.8)	0.6

≥2	42 (40-44)	0.90 (0.72-1.2)		1.0 (0.62-1.8)	1.1 (0.63-1.7)	0.9
History of previous tuberculosis (%; 95%CI)	11 (9.6-13)	1.7 (1.2-2.4)	0.84 (0.71-0.99)	2.1 (1.4-3.0)	2.0 (1.4-2.9)	<0.001
Tuberculin skin test result (%; 95%CI): *unknown*	28 (26-30)	0.99 (0.76-1.3)		*Reference*	*Reference*	*Reference*
negative	22 (21-24)	0.58 (0.39-0.86)		0.60 (0.35-0.96)	0.60 (0.35-1.0)	0.05
positive	50 (48-52)	1.1 (0.87-1.3)		1.1 (0.76-1.5)	1.1 (0.74-1.5)	0.8
**INDEX CHARACTERISTICS**						
Age (median; IQR)	26 (20-36)	NA		1.0 (0.99-1.0)	1.0 (0.99-1.0)	0.7
Sex (% male; 95%CI)	60 (58-62)	1.1 (0.90-1.3)	0.73 (0.56-0.94)	1.5 (1.1-2.1)	1.5 (1.0-2.1)	0.03
Sputum smear status (%; 95%CI) *Negative*	2.6 (2.0-3.4)	0.95 (0.75-1.2)[9]		*Reference*[9]	*Reference*[9]	*Reference*[9]
1+	36 (34-38)					
2+	31 (30-34)	0.82 (0.62-1.1)		0.87 (0.60-1.3)	0.87 (0.61-1.3)	0.5
3+	30 (28-32)	1.0 (0.79-1.3)		1.1 (0.75-1.6)	1.1 (0.77-1.5)	0.7
Drug sensitivity (%; 95%CI): *sensitive*	80 (79-82)	0.93 (0.79-1.1)		*Reference*	*Reference*	*Reference*
isoniazid mono-resistant	8.5 (7.3-10)	0.70 (0.39-1.3)		0.75 (0.37-1.4)	0.74 (0.42-1.3)	0.3
multi-drug resistant	11 (10-13)	1.1 (0.75-1.7)		1.2 (0.75-1.9)	1.2 (0.73-2.0)	0.5
Long diagnostic delay (≥ 60 days of symptoms prior to diagnosis) (%; 95%CI)	53 (51-55)	0.96 (0.78-1.2)	0.90 (0.72-1.1)	1.1 (0.78-1.4)	1.1 (0.80-1.5)	0.6
Frequent cough[4] (every few seconds/minutes vs every few hours/no cough) (%; 95%CI)	11 (10-13)	0.91 (0.59-1.4)	0.93 (0.80-1.1)	0.98 (0.58-1.6)	1.0 (0.61-1.6)	0.99
Known to have HIV infection[5] (%; 95%CI)	1.8 (1.3-2.5)	NA			NA	NA
Exposure to index case						
A) Contact hours spent in the same room per day with index case in the 2 weeks preceding index case diagnosis (median hours; IQR)	5 (2-10)	NA		1.03 (1.0-1.05)	1.02 (1.0-1.05)	0.03
B) Prolonged exposure to the index case[6] (≥ 5 hours spent in the same room per day in the 2 weeks preceding index case diagnosis) (%; 95%CI)	53 (51-55)	1.1 (0.94-1.4)	0.71 (0.55-0.90)	1.6 (1.2-2.2)	1.6 (1.2-2.2)	0.003
**HOUSEHOLD CHARACTERISTICS**						
Lower community household socioeconomic position (%; 95%CI)	45 (42-47)	1.1 (0.92-1.4)	0.78 (0.63-0.96)	1.5 (1.1-2.0)	1.4 (1.0-2.0)	0.03
Exposure to indoor air pollution (%; 95%CI)	35 (32-37)	1.3 (1.0-1.6)	0.75 (0.61-0.92)	1.7 (1.2-2.3)	1.7 (1.3-2.4)	0.001
Any other household member known to have previously had tuberculosis[7] (%; 95%CI)	37 (35-39)	1.3 (1.0-1.5)	0.74 (0.60-0.91)	1.7 (1.3-2.3)	1.7 (1.2-2.3)	0.001
C) Windows per room (median windows per room; IQR)	0.67 (0.5-1.0)	NA		0.74 (0.60-0.92)	0.70 (0.51-0.96)	0.03
D) Fewer windows per room[8] (<0.67 windows per room) (%; 95%CI)	40 (37-42)	1.2 (0.96-1.5)	0.76 (0.62-0.94)	1.6 (1.1-2.1)	1.6 (1.1-2.2)	0.006

NA indicates not applicable. BCG indicates bacille Calmette-Guerin. HIV indicates human immunodeficiency virus. IQR indicates interquartile range. 95%CI indicates 95% confidence interval. SD indicates standard deviation. For variables where missing data were less than 2%, the median value was used to estimate the data and measures were then calculated for all participants.

[1]Data were unavailable for 54% of participants who were excluded from this analysis.

[2]Three of the four contacts known to have HIV infection were diagnosed during study follow-up and the time of seroconversion is not known. As HIV testing was not universal we did not consider this variable for inclusion in a risk score due to potential bias.

[3]BMI indicated body mass index and was adjusted for age using the WHO BMI-for-age charts. For those aged 15, 16, 17 or 18 years, BMI was multiplied by 1.12, 1.09, 1.05 and 1.02 respectively. The units of BMI are kg/m^2

[4]Data were self-reported and were unavailable for 14% of participants. The median value was used to estimate the data. Measures were then calculated for all participants.

[5]HIV testing among index cases was not universal and therefore we did not consider this variable eligible for inclusion in a risk score due to potential bias. In analysis using the data available, the hazard ratio for tuberculosis in contacts if the index cases had known HIV infection was 1.7 (p=0.2).

[6]The relationship between hours [(A)] as a continuous variable and tuberculosis incidence was linear and this variable was therefore dichotomised by the median value [(B)]
[7]This variable includes the index case but excludes the current episode of illness affecting the index case
[8]The relationship between windows per room [(C)] as a continuous variable and tuberculosis incidence was linear and this variable was therefore dichotomised by the median value [(D)]
[9]The reference category for sputum smear status was combined as negative and 1+ due to the small number of participants with sputum smear negative tuberculosis

Table S2: Multivariable analysis of factors associated with tuberculosis in the Ventanilla derivation cohort including tuberculin skin test (TST) (n=2,017).

Variable	Unadjusted hazard ratio (95%CI)	Adjusted hazard ratio (95%CI)	p value	Regression coefficient
**CONTACT CHARACTERISTICS**				
BMI	0.87 (0.84-0.91)	0.87 (0.83-0.91)	<0.001	-0.143
History of previous tuberculosis	2.0 (1.4-2.9)	1.7 (1.1-2.5)	0.01	0.514
High risk age group (aged 15-19 or ≥50 years)	1.5 (1.1-2.0)	1.4 (0.98-1.8)	0.06	0.297
TST result[1]: *unknown*	*Reference*	*Reference*	*Reference*	*Reference*
negative	0.60 (0.35-1.0)	0.64 (0.38-1.1)	0.1	-0.440
positive	1.1 (0.74-1.5)	1.1 (0.79-1.6)	0.5	0.127
**INDEX CHARACTERISTCS**				
Prolonged exposure to the index case	1.6 (1.2-2.2)	1.8 (1.3-2.4)	<0.001	0.565
Exposure to a male index case	1.5 (1.0-2.1)	1.7 (1.2-2.4)	0.001	0.543
**HOUSEHOLD CHARACTERISTICS**				
Lower community household socioeconomic position	1.4 (1.0-2.0)	1.3 (0.94-1.8)	0.1	0.267
Exposed to indoor air pollution	1.7 (1.3-2.4)	1.4 (0.98-1.9)	0.07	0.304
Any other household member known to have previously had tuberculosis	1.7 (1.2-2.3)	1.6 (1.2-2.2)	0.002	0.488
Fewer windows per room	1.6 (1.1-2.2)	1.6 (1.2-2.2)	0.004	0.470

A full description of these variables, and the rationale for their definition, can be found in appendix pages 1-6. BMI indicates body mass index. 95%CI indicates 95% confidence interval. Test of proportional hazards assumption for the entire model: x^2=8.02, p=0.71

[1]In analysis only including people with a known TST result, the unadjusted hazard ratio for tuberculosis for having a positive TST result compared to a negative TST result was 1.8 (95%CI: 1.1-2.9; p=0.02) and the hazard ratio adjusted for all other variables in the model was 1.8 (95%CI: 1.1-3.0, p=0.02).

Figure S1a: Annual tuberculosis incidence rate following exposure among all contacts in the Ventanilla derivation cohort (n=2,017) plotted against community case notification rate 2002-2014

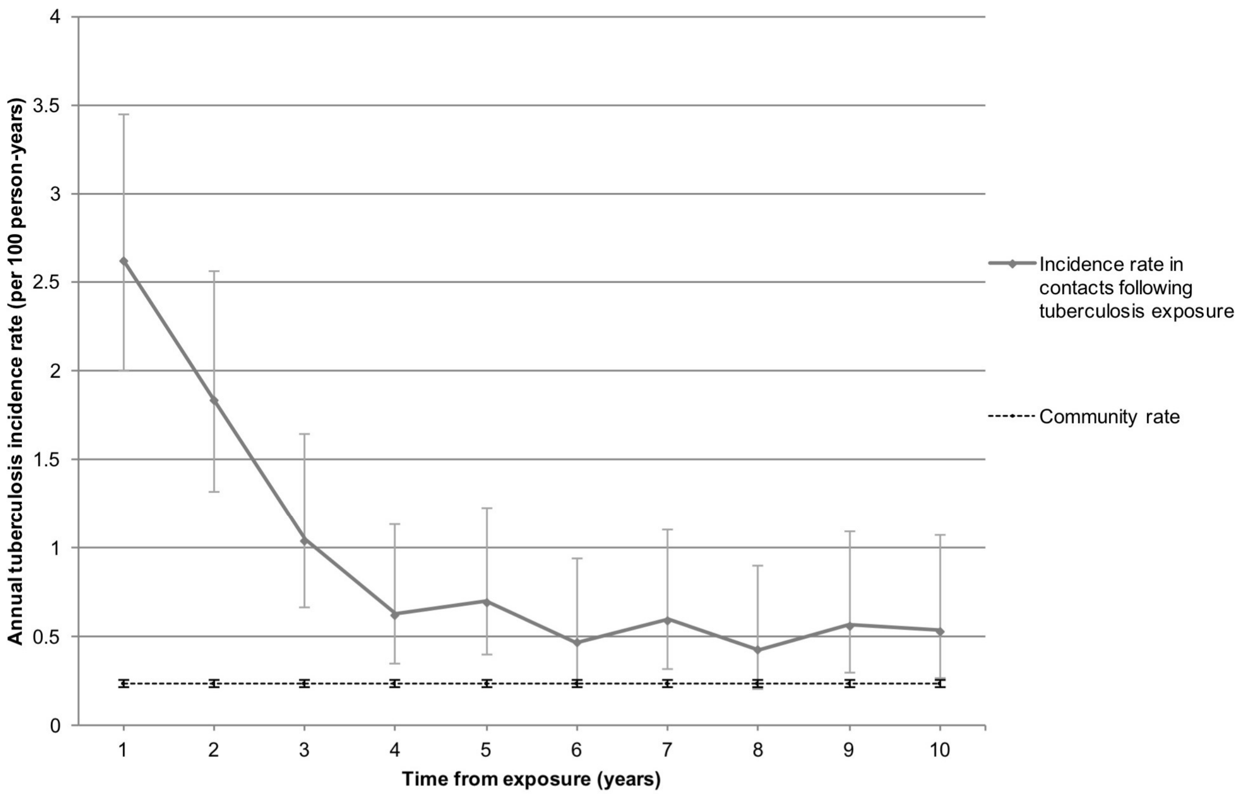

Figure S1b: Trends in tuberculosis incidence among contacts in the Ventanilla derivation cohort (n=2,017) following exposure stratified by risk score group as low risk (n=601), medium risk (n=881) and high risk (n=535)

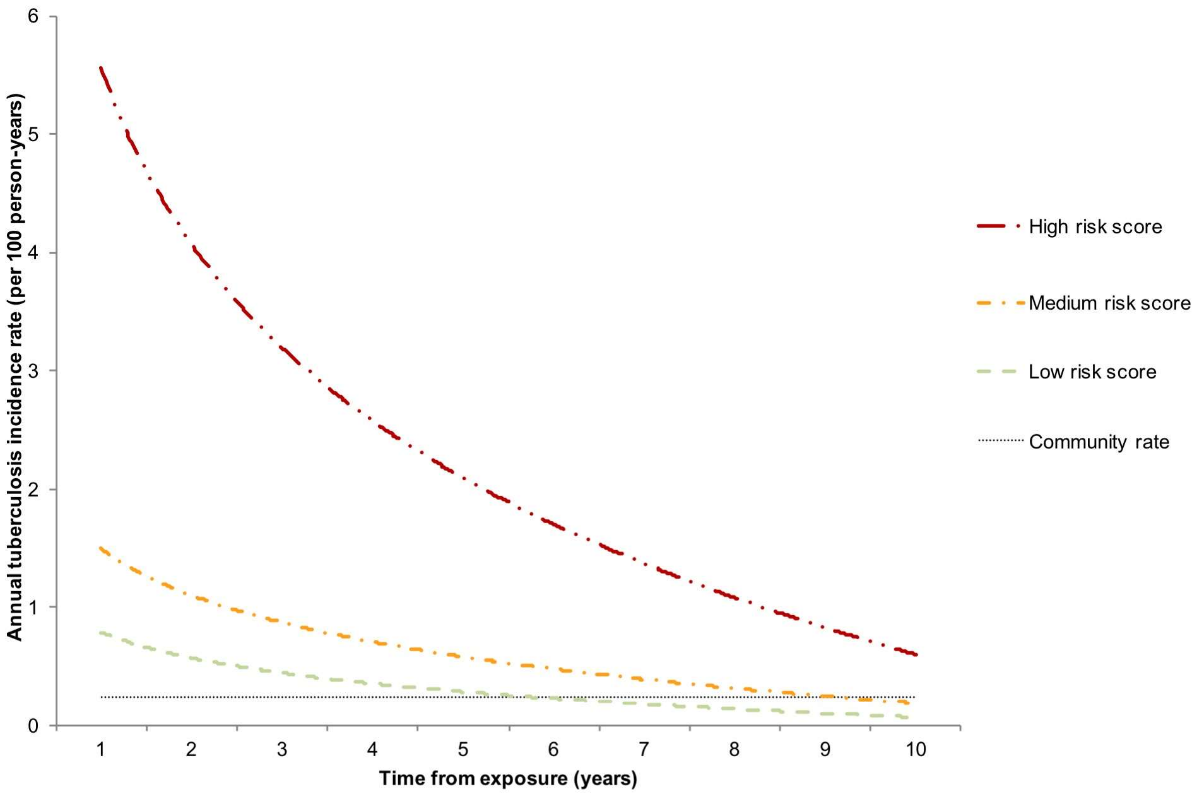

In Figure 1a the error bars represent 95% confidence intervals. In Figure 1b, each risk group's incidence rates were smoothed by fitting an exponential decline curve. The r^2 values for the log-transformed rates in the low, medium, and high risk groups are 0.68, 0.57, and 0.84 respectively. Community rate is defined in the methods.

Figure S2: 10-year predicted risk of tuberculosis derived from the exact cox-regression model (dotted line) versus 10-year observed risk of tuberculosis derived from Kaplan-Meier functions (solid bars) for risk score deciles of population and pre-defined risk score groups in the Ventanilla derivation cohort (n=2,017).

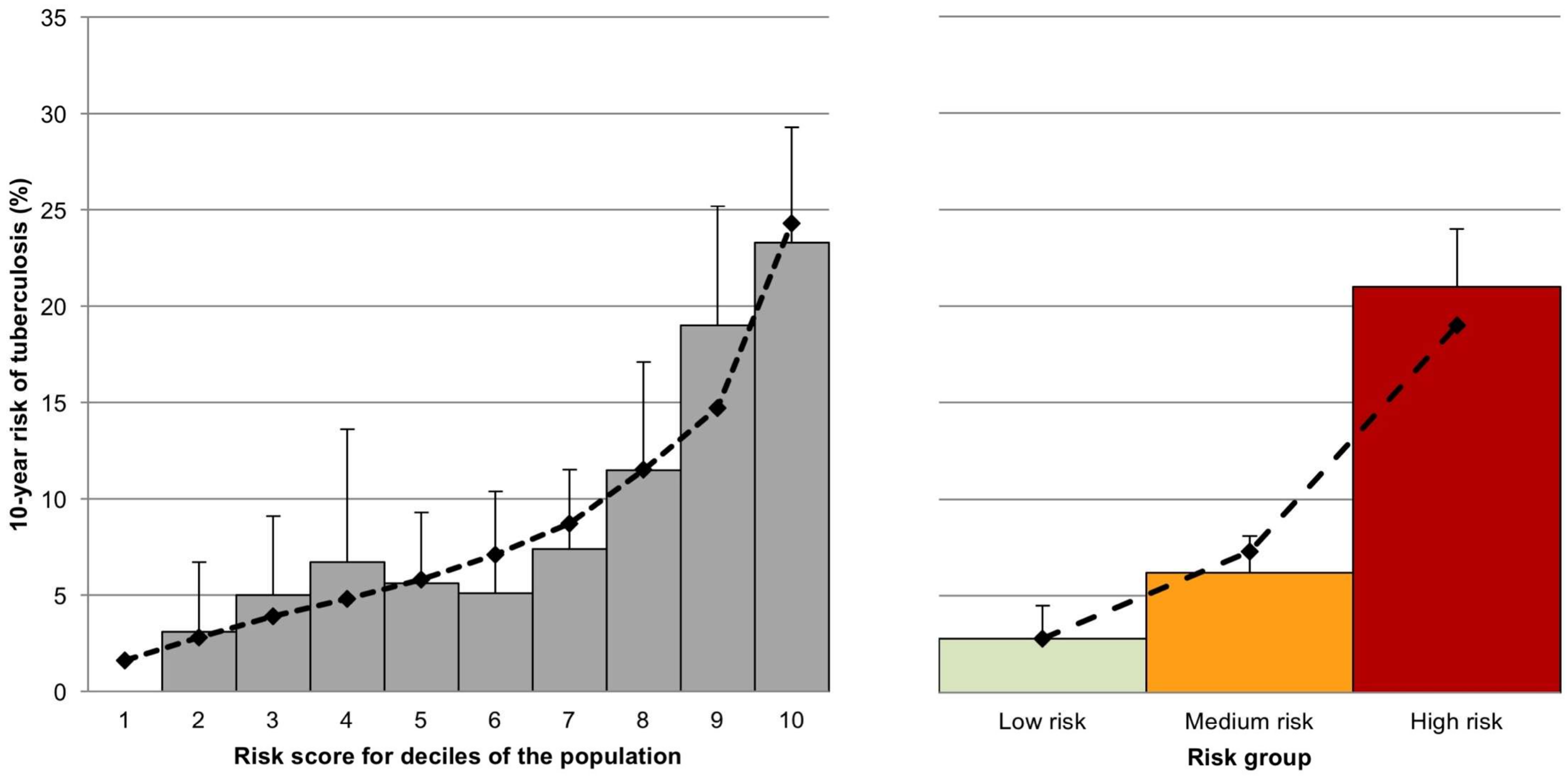

	1	2	3	4	5	6	7	8	9	10	Low risk	Medium risk	High risk
**Absolute proportion of population in decile or group (%)**	9.3	10	10	5.4	13	7.2	13	10	9.2	12	30	44	27
**Cumulative proportion of population (%)**	9.3	19	30	35	48	55	69	79	88	100	30	74	100
**Absolute proportion of all TB diagnoses in decile or group (%)**	0.6	3.4	5.6	4.5	7.9	4.5	11	12	19	32	10	30	60
**Cumulative proportion of TB diagnoses (%)**	0.6	4.0	9.6	14	22	27	37	49	68	100	10	40	100

For the purpose of this chart, the integer risk score was split into ten deciles of approximately equal population size. 10-year predicted risk was calculated for each contact using the exact regression coefficients from the Cox model and the mean in each decile/group taken. The 10-year observed risk within each decile/group was derived from Kaplan-Meier functions.

Figure S3: Cumulative risk of tuberculosis among contacts stratified by risk score group. Error bars represent 95% confidence intervals. The data tables present observed risk of tuberculosis at specific time points derived from Kaplan Meier functions with 95% confidence intervals and the number at risk at each time point with corresponding failure events.

Figure S3a: Ventanilla derivation cohort (n=2,017). C statistic=0.72

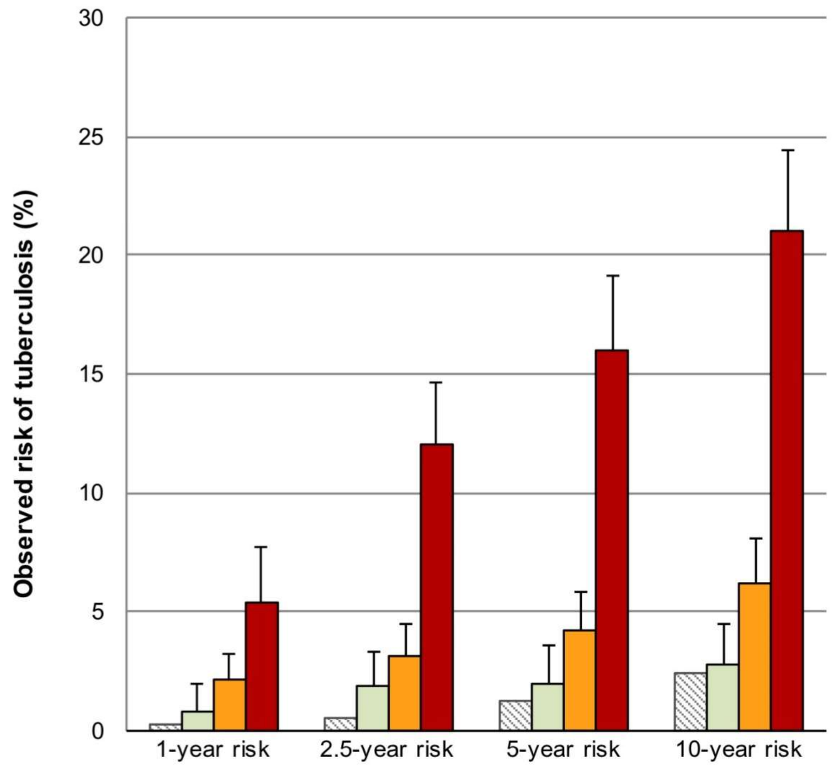

		1-year risk	2.5-year risk	5-year risk	10-year risk
Risk of tuberculosis (95%CI)	High risk score (n=535)	5.4 (3.8-7.7)	12 (9.1-15)	16 (13-19)	21 (17-24)
	Medium risk score (n=881)	2.1 (1.3-3.2)	3.1 (2.1-4.5)	4.2 (3.1-5.8)	6.2 (4.8-8.1)
	Low risk score (n=601)	0.83 (0.35-2.0)	1.9 (1.0-3.3)	2.0 (1.2-3.6)	2.8 (1.7-4.4)
	Community risk	0.24	0.60	1.2	2.4

Number at risk (failure events)	0 years	1 year	2.5 years	5 years	10 years
High risk score	535 (29)	501 (32)	451 (20)	410 (23)	313
Medium risk score	881 (18)	855 (9)	806 (9)	760 (15)	596
Low risk score	601 (5)	594 (6)	558 (1)	537 (4)	401

Figure S3b: Callao validation cohort (n=1,910). C statistic=0.67

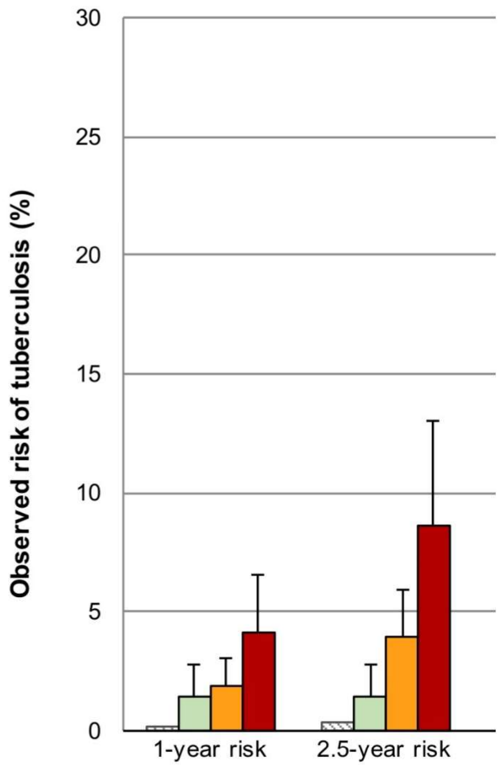

	1-year risk	2.5-year risk
High risk score (n=417)	4.1 (2.6-6.5)	8.6 (5.9-13)
Medium risk score (n=918)	1.9 (1.2-3.0)	3.9 (2.5-5.9)
Low risk score (n=575)	1.4 (0.70-2.8)	1.4 (0.70-2.8)
Community risk	0.17	0.43

	0 years	1 year	2.5 years
High risk score	417 (17)	400 (13)	72
Medium risk score	918 (17)	901 (10)	176
Low risk score	575 (8)	567 (0)	112

Community risk is defined in the methods. The C statistics were calculated using the continuous risk score as the predictor variable.

Figure S4: Sensitivity analysis. Cumulative risk of tuberculosis among contacts stratified by risk score group excluding contacts who commenced tuberculosis treatment within 6 months of the index case. Error bars represent 95% confidence intervals. The data tables present observed risk of tuberculosis at specific time points derived from Kaplan Meier functions with 95% confidence intervals and the number at risk at each time point with corresponding failure events.

Figure S4a: Ventanilla derivation cohort (n=2,017). C statistic=0.74

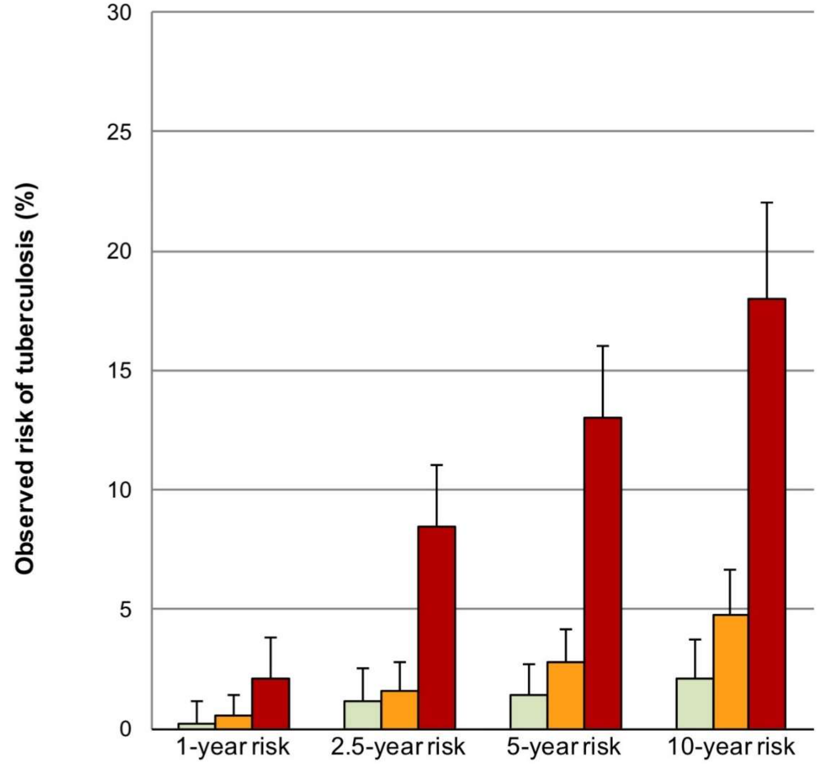

Risk of tuberculosis (95%CI)	1-year risk	2.5-year risk	5-year risk	10-year risk
High risk score (n=514)	2.1 (1.9-3.8)	8.5 (6.4-11)	13 (10-16)	18 (15-22)
Medium risk score (n=866)	0.58 (0.24-1.4)	1.6 (0.98-2.8)	2.8 (1.9-4.2)	4.8 (3.5-6.7)
Low risk score (n=597)	0.17 (0.02-1.2)	1.2 (0.57-2.5)	1.4 (0.69-2.7)	2.1 (1.2-3.7)

Number at risk (failure events)	0.5 years	1 year	2.5 years	5 years	10 years
High risk score	514 (11)	501 (32)	451 (20)	410 (23)	313
Medium risk score	866 (5)	855 (9)	806 (9)	760 (15)	596
Low risk score	597 (1)	594 (6)	558 (1)	537 (4)	401

Figure S4b: Callao validation cohort (n=1,910). C statistic=0.75

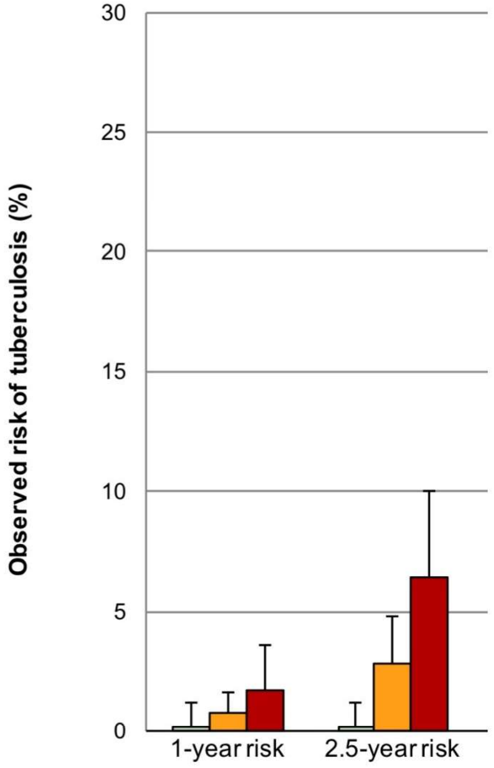

Risk of tuberculosis (95%CI)	1-year risk	2.5-year risk
High risk score (n=407)	1.7 (0.82-3.6)	6.4 (4.0-10)
Medium risk score (n=908)	0.77 (0.37-1.6)	2.8 (1.6-4.8)
Low risk score (n=568)	0.18 (0.02-1.2)	0.18 (0.02-1.2)

Number at risk (failure events)	0.5 years	1 year	2.5 years
High risk score	407 (7)	400 (13)	72
Medium risk score	908 (7)	901 (10)	176
Low risk score	568 (1)	567 (0)	112

The C statistics were calculated using the continuous risk score as the predictor variable.

Figure S5: Calibration chart plotting 2.5-year predicted risk of tuberculosis against 2.5-year observed risk of tuberculosis

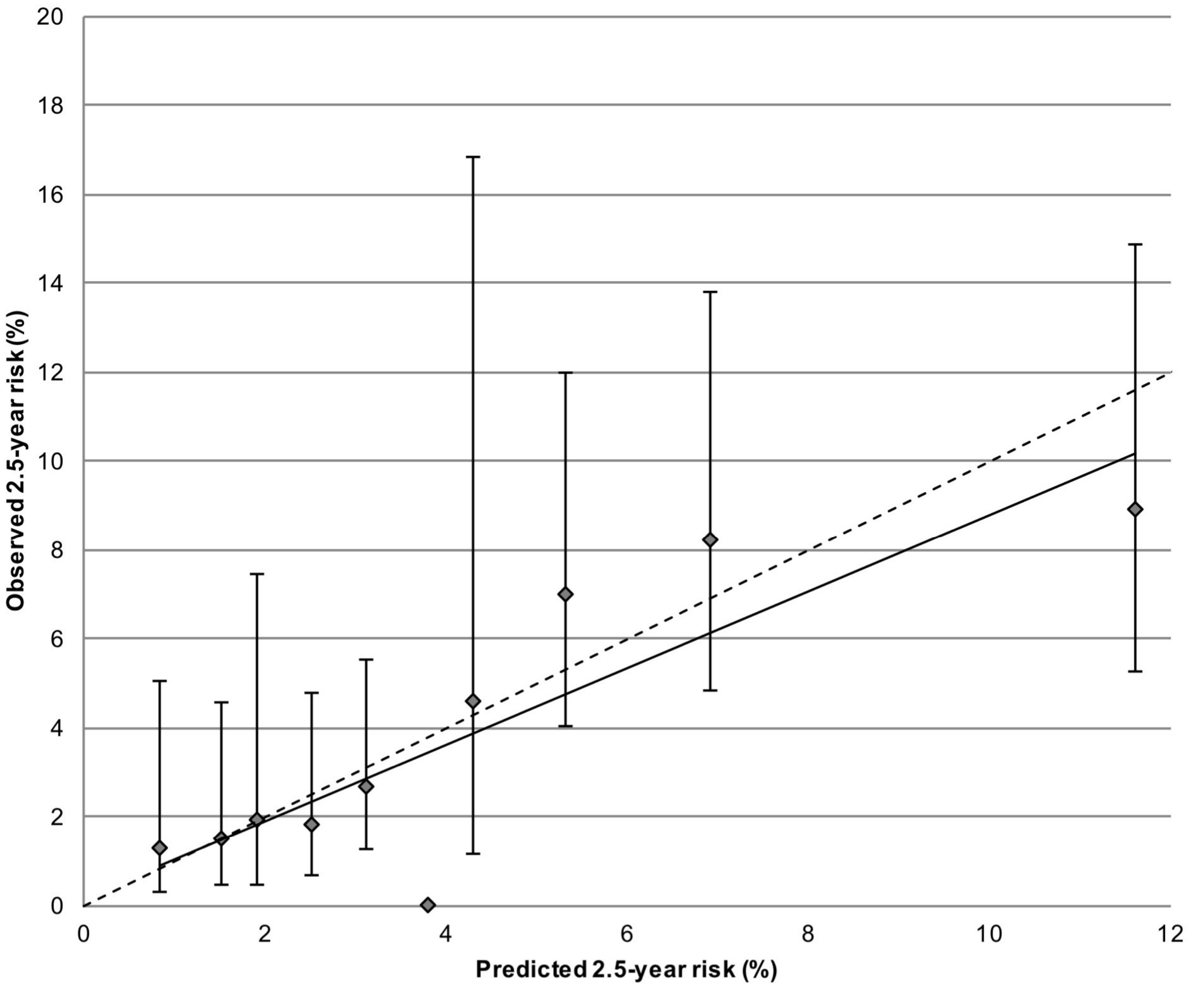

For the purposes of this chart, the integer risk score was split into ten deciles of approximately equal population size which are represented by the plotted points. 2.5-year observed risk within each decile of integer risk score was derived from Kaplan-Meier functions. Mean 2.5-year predicted risk was derived from the original cox model. Error bars represent 95% confidence intervals for observed risk. The dotted line represents perfect calibration, i.e. predicted risk=observed risk. The solid line is a linear trend line for the plotted points. The r^2 for this line was 0.74.

1 Saunders MJ, Tovar MA, Zevallos K, *et al.* Can micronutrient supplementation prevent TB in vulnerable household contacts? A randomised controlled trial. *Int J Tuberc Lung Dis* 2016; **20**: S114.

2 Instituto Nacional de Estadistica e Informatica. Peru: Migración interna reciente y el sistema de ciudades 2002-2007. Lima, 2011.

3 Instituto Nacional de Estadistica e Informatica. Mapa de Pobreza Provincial y Distrital 2013. Lima, 2015.

4 Instituto Nacional de Estadística e Informática. Crecimiento Económico, Población, Características Sociales y Seguridad Ciudadana en la Provincia Constitucional del Callao. Lima, 2016.

5 Situacion de la Epidemia de VIH en el Peru. https://www.minsa.gob.pe/portada/Especiales/2015/vih/matcom/Situacion-Epidemiologica-VIH-2015.pdf (accessed Dec 21, 2016).

6 Rocha C, Montoya R, Zevallos K, *et al.* The Innovative Socio-economic Interventions Against Tuberculosis (ISIAT) project: An operational assessment. *Int J Tuberc Lung Dis* 2011; **15**: S50–7.

7 Martinez L, Arman A, Haveman N, *et al.* Changes in tuberculin skin test positivity over 20 years in periurban shantytowns in Lima, Peru. *Am J Trop Med Hyg* 2013; **89**: 507–15.

8 Moore DA, Evans CA, Gilman RH, *et al.* Microscopic-observation drug-susceptibility assay for the diagnosis of TB. *N Engl J Med* 2006; **355**: 1539–50.

9 Toit K, Mitchell S, Balabanova Y, *et al.* The Colour Test for drug susceptibility testing of Mycobacterium tuberculosis strains. *Int J Tuberc Lung Dis* 2012; **16**: 1113–8.

10 World Health Organization. Growth reference 5-19 years. http://www.who.int/growthref/who2007_bmi_for_age/en/ (accessed April 19, 2016).

11 Hippisley-Cox J, Coupland C, Vinogradova Y, Robson J, May M, Brindle P. Derivation and validation of QRISK, a new cardiovascular disease risk score for the United Kingdom: prospective open cohort study. *BMJ* 2007; **335**: 136.

12 World Health Organisation. TB burden estimates. http://www.who.int/tb/country/data/download/en/ (accessed May 2, 2017).

13 World Health Organisation. Guidelines on the management of latent tuberculosis infection. 2015.

14 Smieja M, Marchetti C, Cook D, Smaill F. Isoniazid for preventing tuberculosis in non-HIV infected persons. *Cochrane Database Syst Rev* 1999; **Issue 1**: Art. No.: CD001363. DOI: 10.1002/14651858.CD001363.

15 Sterling T, Villarine ME, Borisov A, *et al.* Three Months of Rifapentine and Isoniazid for Latent Tuberculosis Infection. *N Engl J Med* 2011; **365**: 683–93.

16 Royston P, Altman DG. External validation of a Cox prognostic model: principles and methods. *BMC Med Res Methodol* 2013; **13**: 33.

17 Steyerberg EW, Harrell FE, Borsboom GJ, Eijkemans M, Vergouwe Y, Habbema JDF. Internal validation of predictive models: Efficiency of some procedures for logistic regression analysis. *J Clin Epidemiol* 2001; **54**: 774–81.

18 Wingfield T, Boccia D, Tovar MA, *et al.* Designing and implementing a socioeconomic intervention to enhance TB control : operational evidence from the CRESIPT project in Peru. *BMC Public Health* 2015; **15**: 1–16.

19 Wingfield T, Tovar MA, Huff D, *et al.* The economic effects of supporting tuberculosis-affected households in Peru. *Eur Respir J* 2016; **48**: 1396–410.

20 Wingfield T, Tovar MA, Huff D, *et al.* A randomized controlled study of socioeconomic support to enhance tuberculosis prevention and treatment, Peru. *Bull World Heal Organ* 2017; **95**: 270–80.
